# Livestock Depredations and Prevention Strategies to Foster Human‐Carnivore Coexistence in Western Mongolia's High Mountain Systems

**DOI:** 10.1002/ece3.74124

**Published:** 2026-07-31

**Authors:** Marcello Franchini, Charleen Gavette, Rodney Jackson, Kayley Bateman, Ashleigh Lutz‐Nelson, Geraldine Werhahn, Yelik Nurbat, Marco Zaccaroni, Fabio Dartora, Munkhtsog Bayaraa, Claudio Augugliaro

**Affiliations:** ^1^ Department of Agricultural, Food, Environmental and Animal Sciences University of Udine Udine Italy; ^2^ Department of Research and Conservation Wildlife Initiative Italia ETS Verona Italy; ^3^ Snow Leopard Conservancy San Francisco California USA; ^4^ Himalayan Wolves Project Switzerland; ^5^ IUCN/SSC Canid Specialist Group, Wildlife Conservation Research Unit University of Oxford Oxford UK; ^6^ Khokh Serkh Special Protected Area's Administration Deluun Sum Mongolia; ^7^ Wildlife Initiative Ulaanbaatar Mongolia; ^8^ Department of Biology University of Florence Florence Italy; ^9^ Institute of Biology, Mongolian Academy of Sciences Ulaanbaatar Mongolia; ^10^ Department of Ecology and Evolution University of Lausanne Lausanne Switzerland

**Keywords:** *Canis lupus*, Foxlights, herder attitudes, human‐carnivore coexistence, Mongolia, *Panthera uncia*

## Abstract

The decline of large carnivores and the overabundance of livestock practices contribute significantly to ecosystem degradation. Large carnivores, through livestock depredations, can cause substantial economic damage leading to resentment among herders and retaliatory killings. Understanding herders' attitudes toward large carnivores and implementing effective non‐lethal prevention measures is thus crucial to enhancing human‐carnivore coexistence. Through structured interviews, we assessed attitudes of 58 herders toward the gray wolf *Canis lupus* and the snow leopard ( *Panthera uncia* ) in the Bayan‐Ölgii province, Mongolia, where livestock practices form the mainstay of livelihood for local communities. Furthermore, we characterized carnivore depredations and tested the use of Foxlights as carnivore deterrents at night. To achieve this goal, 20 herders were randomly selected as ‘treatment’ groups while 18 as ‘control’ groups. Herders' attitudes toward carnivores were explored using a Bayesian cumulative logit mixed‐effects model, while the effectiveness of Foxlights was assessed using Bayesian generalized linear mixed‐effects models, as well as a Bayesian generalized linear model. Our findings revealed that attitudes toward the wolf were entirely negative, while those toward the snow leopard were more positive. In terms of livestock losses, sheep and goats were the most affected by both snow leopards and wolves. However, in relation to availability, yaks and horses were the preferred prey. When comparing ‘treatment’ and ‘control’ groups, the use of Foxlights was associated with an approximately 30% reduction in night‐time depredation events on sheep and goats, that is, *n* = 11 in ‘treatment’ groups versus *n* = 31 in ‘control’ groups. To reduce the magnitude of human‐carnivore conflict it is crucial to encourage collaboration among various institutions and stakeholder groups. Additionally, implementing non‐lethal prevention measures alongside efficient husbandry practices should be encouraged.

## Graphical abstract

The implementation of visual deterrents (i.e., Foxlights) proved to be effective in reducing the number of night‐time livestock depredations by both snow leopards and wolves.

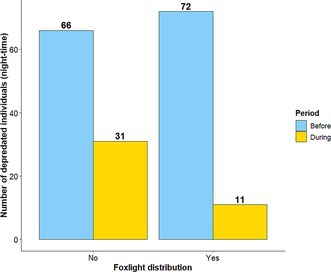

## 1 Introduction

Human‐carnivore conflict represents one of the most pressing sustainable development challenges worldwide, threatening both carnivore persistence (Carter and Linnell 2016; Boronyak et al. 2020; König et al. 2020; Braczkowski et al. 2023) and the continuity of traditional human practices in conflict hotspot areas (Holmern et al. 2007). Reconciling wildlife conservation with the resource needs of a growing human population remains a major global challenge (Woodroffe et al. 2005; Treves et al. 2006; Dickman et al. 2011). In this context, adopting integrated socio‐ecological perspectives to better understand the interlinkages between people and wildlife, as well as the mechanisms that may weaken or strengthen these relationships, is of paramount importance (Carter et al. 2014).

Large carnivores (herein, carnivores), through top‐down ecological effects in the food chain, contribute to regulating the abundance of other species, thereby promoting biodiversity (Estes et al. 2011; Ripple et al. 2014). Likewise, sustainable extensive grazing practices provide ecosystem services to humans, mainly in the form of carbon storage in the soil, nutrient recycling, food (e.g., milk, cheese, meat), and conservation of natural habitats (Battaglini et al. 2014; Salvador et al. 2017; Pachoud et al. 2020). However, the overabundance of livestock contributes to environmental degradation, and the threat posed by carnivores to local livelihoods may undermine conservation efforts (Bagchi and Mishra 2006; Wang and Macdonald 2006), particularly in developing countries, where livestock and agricultural activities are among the primary sources of income for local inhabitants (e.g., Guerisoli et al. 2017; Mijiddorj et al. 2018; Augugliaro et al. 2020).

After the fall of Socialism in 1990, the Mongolian market system shifted from a centralized to an open one (Dorj and Yavuukhulan 2014). As a result, the cashmere trade increased steadily, and the number of livestock, particularly goats *Capra hircus* , followed the same upward trend (National Statistics Office of Mongolia 2022). This trend has led to (i) soil degradation due to livestock trampling behaviors and overgrazing, as well as interspecific competition between wild and domestic ungulates (Berger et al. 2013; Rovero et al. 2020), and (ii) increasing human‐carnivore conflicts (Mijiddorj et al. 2018; Augugliaro et al. 2020). In Mongolia, livestock herding represents one of the mainstays for local communities, and livestock depredations may threaten livestock herders' (herein, herders) economic stability along with the long‐term survival of carnivore populations (Augugliaro et al. 2020). The two main carnivore species affected are the snow leopard ( *Panthera uncia* ) and gray wolf *Canis lupus* (herein, wolf). The snow leopard, with a fragmented distribution across 12 Asian Countries and 7446–7996 estimated individuals (McCarthy and Mallon 2023), is globally classified as “Vulnerable” on the International Union for Conservation of Nature (IUCN) Red List of Threatened Species (McCarthy et al. 2017). Conversely, the wolf, with a distribution covering much of the Holarctic region, is globally classified as a species of “Least Concern” (Boitani et al. 2023). However, in Mongolia, the snow leopard is classified as “Endangered” and protected under national legislation, whereas the wolf, despite being locally classified as “Near Threatened”, can still be legally hunted (Clark et al. 2006). The government not only tolerates wolf hunting, including within protected areas, but in many cases also pays bounties to hunters for each wolf killed (Augugliaro et al. 2020; Werhahn et al. 2025). In Mongolia, the snow leopard has a distribution ranging from Turgen (Uvs province) in the northwest to the Gobi Desert in the southwest (Augugliaro et al. 2019; Bayandonoi et al. 2021; Franchini et al. 2023). Conversely, the wolf has a more homogeneous distribution across the whole country (Boitani et al. 2023). Snow leopards primarily inhabit higher elevations, whereas wolves occupy a broader range of habitats across varying elevation gradients, roaming from upper plateaus to valley bottoms. Both carnivore species contribute to livestock losses to varying degrees in the Mongolian Southern Gobi (Mijiddorj et al. 2018), the Tost Mountains (Samelius et al. 2021), and Bayan‐Ölgii Province (Augugliaro et al. 2020). For example, Mijiddorj et al. (2018) reported that herders considered carnivores to be one of the main causes of livestock losses across seasonal periods during a one‐year study (January–December 2010), with wolves believed to be responsible for 1141 losses and snow leopards for 740 losses. Similarly, Augugliaro et al. (2020) found that, over the period October 2016 to September 2017, and after accounting for mortalities due to natural causes (e.g., disease and harsh winters; *n* = 3961 losses), carnivore depredation represented a major cause of livestock losses. Wolves were reported as responsible for 3583 losses, while snow leopards accounted for 167 losses, with no clear seasonal variation observed. The volume of these losses negatively influences people's attitudes and perceptions toward these species, thereby affecting the long‐term prospects for coexistence.

Globally, local attitudes and perceptions of the impacts of carnivores on human activities are key drivers of human‐carnivore conflicts (Dressel et al. 2015; Nyhus 2016; Franchini et al. 2021). Attitudes toward carnivores are influenced by factors such as the herder's sex, age, level of education, social and community attitudes, loss rates and direct experiences with carnivores (Dickman 2010; Alexander et al. 2015; Dressel et al. 2015; Franchini et al. 2021, Franchini, Sartor, et al. 2025; Franchini, Švajda, et al. 2025), oral traditions and storytelling (Álvares et al. 2011; Krange et al. 2017), as well as religious beliefs (Li, Wang, et al. 2013). Furthermore, herders' attitudes toward carnivores can be influenced by the magnitude of damage these animals inflict on livestock (Mijiddorj et al. 2018; Augugliaro et al. 2020). Among affected livestock, sheep *Ovis aries* and goats, due to their smaller body size and limited antipredatory strategies, are more frequently targeted than other species (e.g., Mijiddorj et al. 2018; Augugliaro et al. 2020; Janeiro‐Otero et al. 2020; Parchizadeh et al. 2024). Moreover, with respect to wolves, social status may significantly influence impacts on human livelihoods, as solitary individuals are more prone to attack livestock than pack‐living wolves (Meriggi et al. 1996; Meriggi and Lovari 1996; Linnell et al. 1999; Imbert et al. 2016; Mayer et al. 2022). Given this conflictive scenario, further research aimed at identifying the main conditions explaining herder attitudes toward carnivores in Mongolia and the implementation of proper prevention measures is paramount to prioritizing conservation interventions.

Non‐lethal prevention measures can strike a good balance between preserving human activities and conserving carnivores in shared landscapes (Van Eeden et al. 2018). In pastoral landscapes, livestock are exposed to carnivore depredations across both diel periods. However, night‐time represents a particularly vulnerable phase due to reduced human supervision. As a result, many preventive tools, including visual deterrents, are primarily designed to reduce risk during nocturnal hours when active herding is limited. Foxlights are solar‐powered visual deterrents that activate at night, produce intermittent flashing lights of different colors projected at 360°, and are visible up to a km or so. The effectiveness of this method for deterring carnivores has already been explored in other countries (Lesilau et al. 2018; Ohrens et al. 2019; Naha et al. 2020). For example, Lesilau et al. (2018) recorded a significant reduction in livestock depredations by lions ( *Panthera leo* ) following the implementation of Foxlights in Kenya, while Naha et al. (2020) observed a similar trend for livestock depredations by common leopards ( *Panthera pardus* ) in the western Himalaya. However, the effectiveness of this method may vary depending on the carnivore species. For instance, Ohrens et al. (2019) found that visual deterrents were effective against pumas ( *Puma concolor* ) but less so for Andean foxes ( *Lycalopex culpaeus* ), suggesting that the light devices might have attracted foxes or, by deterring pumas, created opportunities for fox visitation. These findings indicate that the effectiveness of visual deterrents is context‐ and species‐dependent. The advantages of implementing Foxlights include their low cost, minimal maintenance compared to other prevention measures (e.g., electrified fences), and ease of relocation, which is particularly useful given the nomadic livestock system adopted by herders in western Mongolia (Augugliaro et al. 2020). Nevertheless, to the best of our knowledge, this is the first time this method has been tested in this area.

The objectives of this research were thus to:

1. Investigate herder attitudes toward snow leopard and wolf. We hypothesized that factors such as age, level of education, number of small and/or large livestock owned, adopted livestock management system and direct experiences with carnivores play a key role in shaping herder attitudes (e.g., Alexander et al. 2015; Dressel et al. 2015; Franchini et al. 2021; Franchini, Sartor, et al. 2025; Franchini, Švajda, et al. 2025). Considering the generally higher impact that wolves exert on livestock (Mijiddorj et al. 2018; Augugliaro et al. 2020), we predicted herders would display more negative attitudes toward wolves than toward snow leopards.
2. Characterize wolf and snow leopard depredation events and prey preference. We hypothesized that wolves, with their apparently greater ecological plasticity, are more inclined to approach and visit human areas in search of food by day or night (e.g., Di Bernardi et al. 2025; Frangini et al. 2025). Snow leopards, by contrast, are more secretive and rarely seen. Thus, we predicted to observe a carnivore‐specific magnitude of conflict, with the frequency of livestock depredations by wolves being significantly greater than that by snow leopards. We further hypothesized that sheep and goats, given their smaller body size and limited antipredatory strategies (e.g., reduced vigilance), represent more easily catchable prey (e.g., Mijiddorj et al. 2018; Augugliaro et al. 2020; Janeiro‐Otero et al. 2020; Parchizadeh et al. 2024). Therefore, relative to livestock availability, we predicted higher depredation rates on sheep and goats than on other livestock species.
3. Characterize wolf depredation events with regards to their social status (i.e., solitary, pair, pack). We hypothesized that wolf social status represents a key determinant in influencing livestock attacks (Meriggi et al. 1996; Meriggi and Lovari 1996; Linnell et al. 1999; Imbert et al. 2016; Mayer et al. 2022). Accordingly, we predicted higher rates of livestock depredation by solitary wolves compared to pairs and/or packs.
4. Provide a preliminary assessment of the potential effectiveness of Foxlights in the area, by comparing night‐time depredation events by carnivores in ‘treatment’ and ‘control’ groups. We hypothesized that visual deterrents are effective at deterring carnivores at night (Lesilau et al. 2018; Ohrens et al. 2019; Naha et al. 2020). Therefore, we predicted to observe significantly fewer livestock depredations by wolves and snow leopards in ‘treatment’ groups.

## 2 Material and Methods

### 2.1 Study Area and Livestock Herding System

**FIGURE 1:**
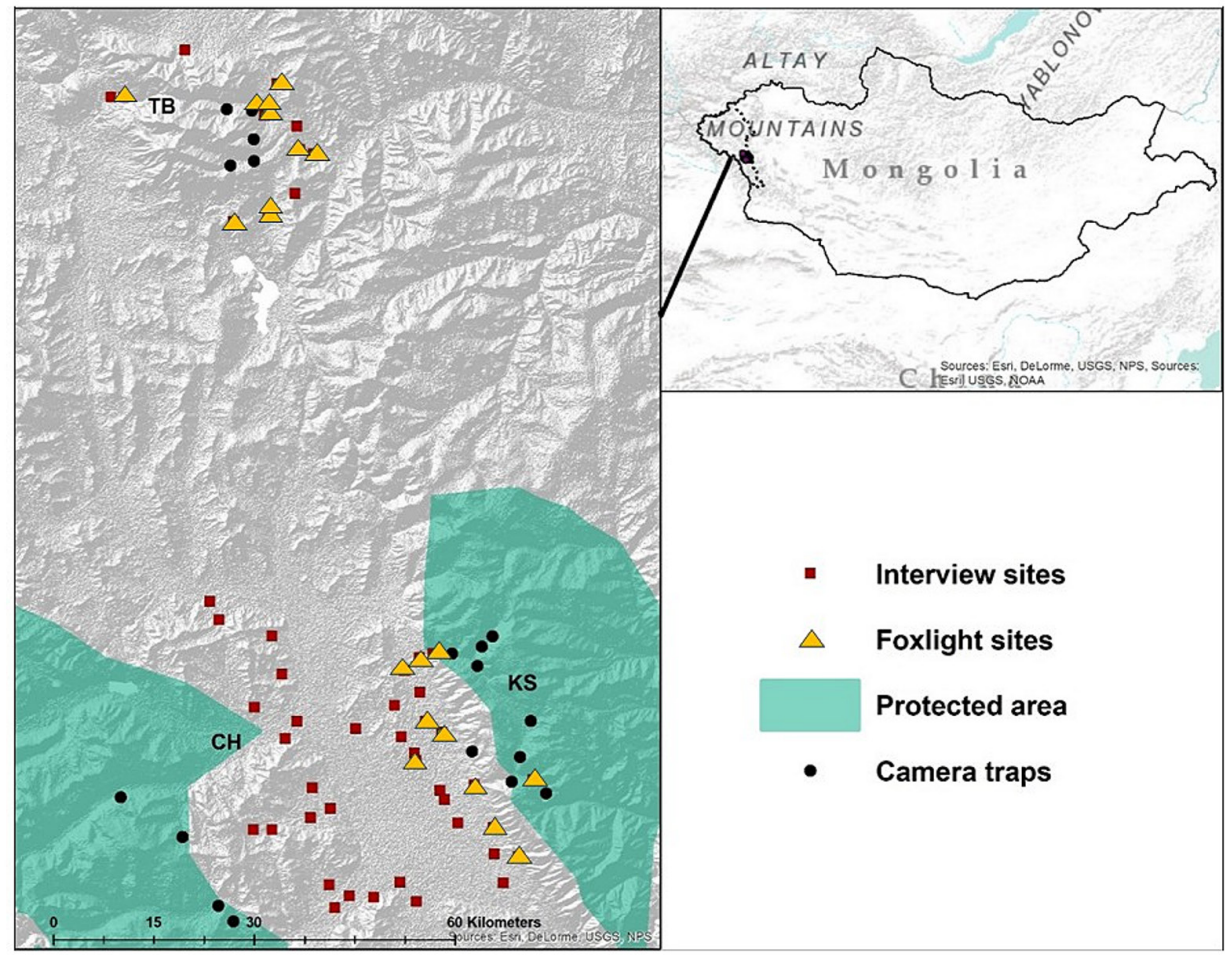
Location of the study area (inset map) and localities (TB = Tolbo, CH = Chigertei, KS = Khokh Serkh) in which interviews (red squares) were conducted, and camera traps (black dots) were placed. In the localities of TB and KS only, red squares also indicate ‘control’ groups. Conversely, ‘treatment’ groups are highlighted with golden triangles.

**FIGURE 2:**
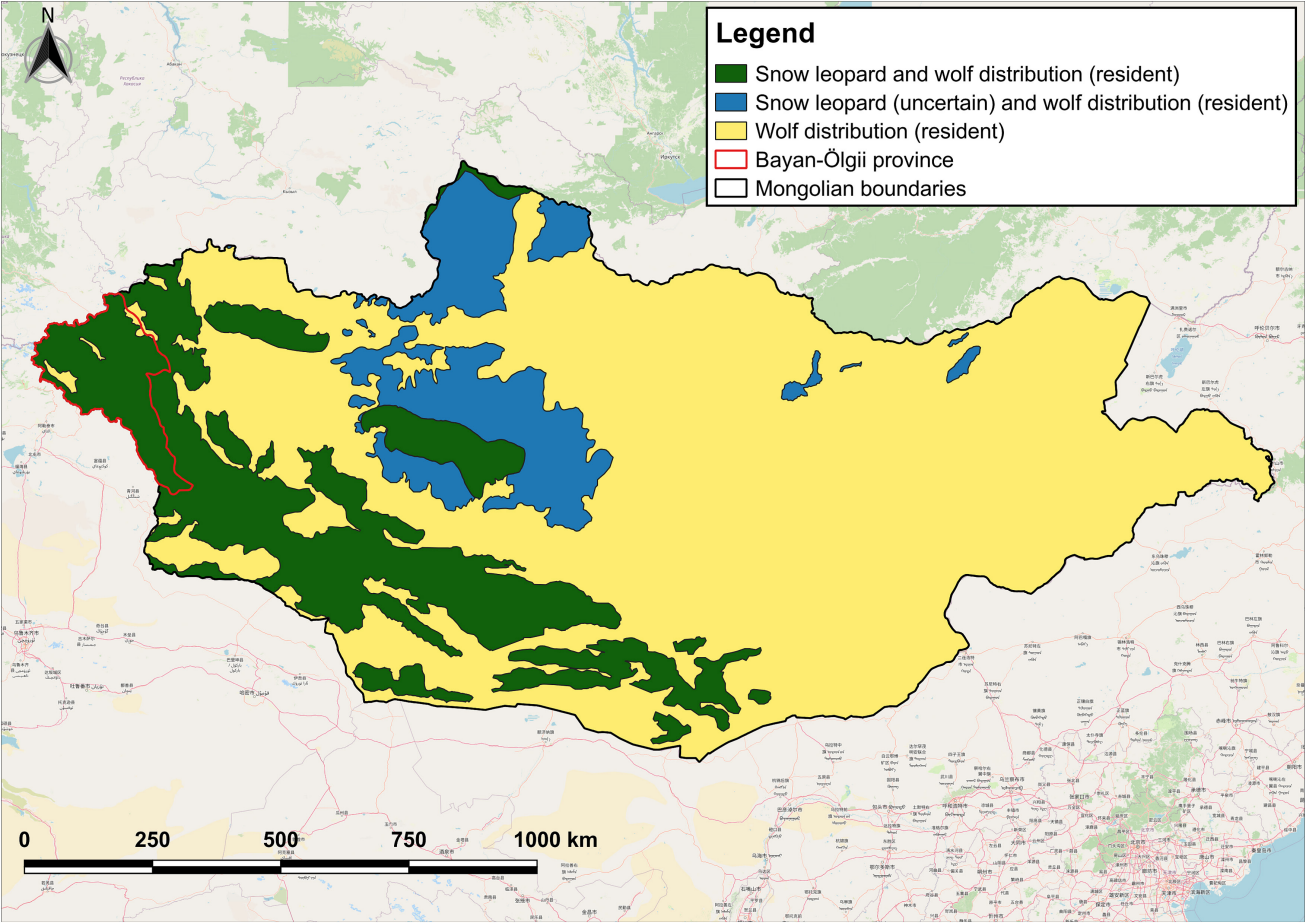
Snow leopard (IUCN 2017) and wolf (IUCN 2023) range of distribution across Mongolia and in the Bayan‐Ölgii province.

The Bayan‐Ölgii province (~45,705 km^2^) is Mongolia's westernmost province, bordering Russia to the north and China to the south (Figure 1). The area falls within the Altay‐Sayan Ecoregion and hosts Mongolia's highest mountain, Khuiten peak, reaching 4374 m above sea level (a.s.l.). The landscape is primarily covered by steppe and alpine meadows, with only 10% of the land covered by forest, mainly Siberian larch ( *Larix sibirica* ) (Augugliaro et al. 2020). The carnivore community encompasses brown bear *Ursus arctos* , wolf, red fox ( *Vulpes vulpes* ), snow leopard, Eurasian lynx ( *Lynx lynx* ), Pallas's cat ( *Otocolobus manul* ), beech marten ( *Martes foina* ), and steppe polecat ( *Mustela eversmanii* ). Among these species, the wolf and the snow leopard mostly overlap across the western area (Figure 2). Conversely, the brown bear and Eurasian lynx occur at very low densities and thus have the sparsest distribution. Potential prey species for the mentioned carnivores are the argali sheep *Ovis ammon* , red deer *Cervus elaphus* , Siberian ibex *Capra sibirica* , Arctic hare *Lepus timidus* , marmots (*Marmota* spp.), pika (*Ochotona* spp.), and Tolai hare *Lepus tolai* (Shehzad et al. 2012; Batsaikhan et al. 2014).

In the Bayan‐Ölgii province, 66,047 of its 105,090 inhabitants live in rural areas (Lkhagvadorj et al. 2013; Augugliaro et al. 2020). Herders are nomadic and relocate seasonally based on local customs, knowledge of pasture conditions, and forage availability. About 2,166,910 livestock are bred in the province, representing 3.3% of the total livestock in Mongolia (National Statistics Office of Mongolia 2022). This includes 1,910,000 sheep and goats, 155,910 cattle *Bos taurus* and yaks *Bos grunniens* , 95,350 horses *Equus ferus* , and 5650 domestic Bactrian camels *Camelus bactrianus* . The herding practices vary depending on the livestock species involved. Sheep and goats are guarded by herders and/or livestock guardian dogs (LGDs) during day‐time and enclosed in corrals (i.e., walls made of stones about 1.3 m high) or kept close to the campsite at night to prevent carnivore attacks. Conversely, cattle, yaks, horses, and domestic Bactrian camels are left to graze freely and unattended both day and night (Augugliaro et al. 2020). As for the adopted LGDs, the Mongolian Bankhar dog is the most frequently used by herders (typically, one or two dogs per household). Mongolian Bankhar dogs are widely valued for their intelligence and perseverance, even under harsh weather conditions. In addition, they are loyal and affectionate toward their families, yet very effective against intruders, including humans, wolves, and snow leopards (Lieb et al. 2021).

### 2.2 Research Project and Data Collection

The overall purpose of the research project was to mitigate human‐carnivore conflict in the Bayan‐Ölgii province by fostering long‐term coexistence practices between people and carnivores, while also exploring the magnitude and characteristics of livestock depredations and human attitudes toward both the snow leopard and wolf. The project officially began in September 2020 and concluded in October 2021. At the beginning of the project, herders were asked to complete a weekly form reporting all information related to depredation events (Supporting Information S1), and Foxlights were randomly distributed among herders. All herders (both equipped and not equipped with Foxlights) participated in livestock depredation data collection. Hereafter, the term “project period” refers exclusively to the 12‐month Foxlights implementation phase (September 2020–October 2021), whereas “pre‐project period” refers to the 12 months preceding implementation (September 2019–August 2020).

#### 2.2.1 Herders' Attitude Toward Snow Leopard and Wolf in the Pre‐Project Period

We conducted structured face‐to‐face interviews using a standardized questionnaire administered to a randomly selected sample of herders (see *Results*) drawn from a complete list of households provided by local administrative records, ensuring balanced representation across the considered three localities: Chigertei, Khokh Serkh, and Tolbo (Figure 1). The structured questionnaire was developed and subsequently reviewed by local collaborators to ensure clarity and cultural appropriateness. Minor adjustments were made after pilot testing with three local herders who were not included in the final sample to improve question clarity and cultural appropriateness. Herder attitudes toward carnivores were explicitly assessed as part of the third section of the questionnaire. The concept of “attitude” was translated into Mongolian using locally meaningful expressions referring to personal evaluation and tolerance toward the species, and interviewers ensured that respondents clearly understood the question before answering. Interviews were conducted in Mongolian by Yelik Nurbat of the Khokh Serkh Special Protected Area Administration, a trained local officer familiar with the socio‐cultural context, which further minimized misinterpretation. The responses obtained from the interviews were subsequently translated into English. The questionnaire was divided into three main sections: (i) the first section, included general questions aimed at collecting information on respondents' characteristics (e.g., age, sex, level of education, job); (ii) the second section, contained questions referring to the adopted livestock management system (e.g., number of livestock bred divided by species, number of days spent outdoor during a year, adopted grazing system), livestock losses (i.e., depredation, other causes), and implemented preventive measures (e.g., presence and number of LGDs); (*iii*) the third section was dedicated to explore herder attitudes toward snow leopards and wolves (see Supporting Information S2). To maximize the likelihood of obtaining honest, reliable and unbiased responses, the possibility of accessing visual deterrent tools was not mentioned during the interviews, and the interviews were conducted without the interviewer expressing any personal opinions or judgments that could influence respondents' answers. Moreover, cameratrapping, together with sign transects and expert knowledge, was used to support the identification of carnivore presence across the study area for interview targeting. Herder attitudes toward the wolf and the snow leopard (i.e., *Please indicate your attitude toward the following species*—Supporting Information S2) was initially assessed through a 5‐point Likert‐scale questionnaire (i.e., 1 = strongly dislike, 2 = slightly dislike, 3 = indifferent, 4 = slightly like, 5 = strongly like). However, with regards to the snow leopard, extreme categories (“strongly dislike” and “strongly like”) were selected by very few respondents, with most answers concentrated in the intermediate categories (2, 3, 4). Given the limited sample size (*n* = 58, see *Results*) and sparse frequencies in extreme levels, retaining all five categories would have resulted in unstable parameter estimates in ordinal models. We therefore collapsed adjacent categories, that is, 1 = dislike (combining strongly dislike and slightly dislike), 2 = indifferent, and 3 = like (combining slightly like and strongly like), to improve model stability, a commonly adopted practice in ecological modeling (Bolker 2008). Importantly, this recoding did not alter the overall pattern of responses. For wolves, responses were consistently negative across respondents, indicating very limited variation in attitude. Thus, collapsing categories did not affect interpretation, as the overall pattern was unequivocally negative.

#### 2.2.2 Livestock Depredations and Prey Preference Analyses in both the Pre‐Project and Project Periods

Additionally, to assess herders' ability to recognize carnivores and identify signs of their presence, we adopted the approach proposed by Augugliaro et al. (2020). Specifically, at the beginning of the project, herders were trained through field‐based activities and the use of visual materials, including photographs depicting typical signs such as footprints, scrapes, and scats, to enhance their capacity to detect carnivore presence. Regardless of whether depredation events occurred, herders were trained to conduct transect surveys from their campsites to grazing areas, with lengths varying from about 500 m to up to 3 km depending on the distance from the campsite to the grazing area. These surveys were designed to help herders identify signs of carnivore presence. When a depredation event occurred (during both day and night), herders were trained to inspect the surroundings of the kill point, searching for signs of carnivore presence. The data collected during in situ kill‐site inspections included: (i) the locality, (ii) the date of observation, (iii) the killed livestock species, (iv) the age class of the attacked livestock, (v) the number of depredated individuals, (vi) the responsible carnivore (identified based on signs such as footprints or scrapes), and (vii) the number of carnivores (identified by footprints, with wolves categorized as solitary individuals, pairs, or packs). Depredation events were distinguished from mortality due to other causes by examining carcasses for the presence of throat and/or limb bite marks. The age class of the attacked livestock species was classified as follows: sheep and goats (< 2 years = young/sub‐adults; ≥ 2 years = adults), yaks (< 4 years = young/sub‐adults; ≥ 4 years = adults), horses (< 6 years = young/sub‐adults; ≥ 6 years = adults). Based on camera trap data and information provided by local herders, feral dogs are either absent or present at very low densities in the area. Moreover, the few roaming individuals are reportedly limited to preying on marmots and lagomorphs. Therefore, the potential bias in attributing depredation events to wild carnivores, particularly wolves, is considered negligible.

#### 2.2.3 Foxlights' Distribution between ‘Treatment’ and ‘Control’ Groups in the Project Period

In the project period, Foxlights were distributed by differentiating between ‘treatment’ and ‘control’ groups. Of the three study localities (Figure 1), Chigertei, based on camera trap data, sign transects, local herders' experience, and protected area officers' knowledge, experienced the fewest carnivore depredations, likely due to a lower density of carnivores in the area. Consequently, Tolbo and Khokh Serkh were those locations in which Foxlight ‘treatment’ vs. ‘control’ groups were placed. Overall, 20 herders were randomly selected as ‘treatment’ group and were each equipped with Foxlights (*n* = 12 in Khokh Serkh, and *n* = 8 in Tolbo), while 18 herders were randomly selected as ‘control’ group and then not equipped with Foxlights (*n* = 10 in Tolbo, and *n* = 8 in Khokh Serkh). Each treatment site was equipped with one Foxlight device, which remained in place continuously throughout the entire study period (12 months). A randomized approach was chosen as it helps in mitigating the most common form of selection bias, which can occur when researchers unconsciously seek to observe desired effects of a ‘treatment’ (Treves et al. 2019). In the area, all herders live in the valley at the same altitudinal gradient, and both the ‘treatment’ and ‘control’ groups in either Tolbo or Khokh Serkh were located at an approximate average distance of 10 km from each other to guarantee the independence of each location. Foxlights were tested exclusively to protect sheep and goats, as these are the only livestock species that are enclosed into corrals or kept close to the campsite at night. Following other research protocols (Ohrens et al. 2019; Naha et al. 2020), Foxlights were installed along the edges of livestock corrals or at the perimeter of campsites (for those herders not possessing corrals; see Table 1), typically placed at a height of 3–4 m from the ground to increase their visibility from a distance, and were rotated to another side of the corral approximately every three weeks to reduce carnivore habituation during the whole study period (i.e., September 2020–October 2021). As part of the nomadic system, herders typically move between 1 and 5 km and reinstall Foxlights each time they relocate. The Foxlights, which randomly emit three different colored flashes, operated automatically each night from sunset to sunrise under pre‐programmed settings. To confirm visitation by snow leopards and/or wolves during the night, each herder inspected the perimeter of corrals and the campsite each morning.

**TABLE 1:** Socio‐economic characteristics of the herders interviewed in Chigertei (*n* = 20), Khokh Serkh (*n* = 20), and Tolbo (*n* = 18).

Socio‐economic characteristics		*n*	%
Age class	20–40	12	20.7
	41–60	38	65.5
	61–80	7	12.1
	> 80	1	1.7
Sex	Male	57	98.3
	Female	1	1.7
Education level (years in school)	1–5	22	37.9
	6–10	34	58.6
	> 10	2	3.4
Number of family members	1–5	17	29.3
	6–10	38	65.5
	> 10	3	5.2
Number of times the family moves in a year	2	1	1.7
	3	9	15.5
	4	47	81
	6	1	1.7
Total small‐sized livestock per family	1–250	19	32.8
	251–500	33	56.9
	> 500	6	10.3
Total large‐sized livestock per family	1–25	27	46.6
	25–50	23	39.7
	> 50	8	13.8
Presence of corral	Yes	54	93.1
	No	4	6.9
Number of livestock guardian dogs	0	16	27.6
	1	38	65.5
	2	4	6.9

### 2.3 Statistical Analysis

Statistical analyses were conducted using the R Software (v. 4.3.2) (R Core Team 2023) with the significance level set at 0.05 for all frequentist statistical tests.

#### 2.3.1 Herders' Attitude toward Snow Leopard and Wolf in the Pre‐Project Period

Herder attitude toward the snow leopard were analyzed exclusively, as all respondents exhibited a negative attitude toward the wolf (see *Results*). To encourage better parameter estimates starting from a relatively small sample size (e.g., Filacorda et al. 2022; Franchini et al. 2022; Gelman and Hill 2006; Gelman et al. 2025; Bikembo et al. 2026), attitudes were examined using Bayesian cumulative logit mixed‐effects model (BCMM) with a *cumulative (link = “logit”)* family, using the ‘brms’ R package (Bürkner 2021). This model was run with 12,000 iterations, four chains, and 2000 warmups. Model convergence was thereafter assessed based on the Rhat value; a diagnostic measure commonly used for assessing the convergence of Markov Chain Monte Carlo (MCMC) simulations (Gelman et al. 2004; Kruschke 2014). As the Rhat value was equal to 1, satisfactory convergence could be inferred (Gelman et al. 2004; Kruschke 2014). The *goodness‐of‐fit* of the BCMM was further evaluated using both the normal probability plot (QQ plot) of residuals and the chains' trace plot (Figures S1 and S2—Supporting Information S3) through the ‘bayesplot’ R package (Gabry and Mahr 2024). Covariates/predictors, that is, age (continuous variable), education level, number of small (sheep, goat) and large (cattle, yak, horse) livestock bred per family, number of LGDs, were included as fixed factor in the BCMM. The interview location was instead introduced as a random factor in the model to account for the variability among groups. We interpreted effects based on the posterior distribution of parameters. When the 95% credible interval did not overlap zero, we considered there to be strong evidence for a directional effect (Gelman and Hill 2006; Gelman et al. 2025). Specific hypotheses for the predicted effect of each covariate/predictor on herders' attitude toward the snow leopard were reported in Table 2.

**TABLE 2:** Hypotheses of the predicted effect of each covariate/predictor on herders' attitude toward the snow leopard.

Covariate/predictor	Predicted effect on attitude	Rationale	Supporting literature
Age	−	Increasing age is often associated with a negative attitude toward carnivores.	Suryawanshi et al. (2014); Piédallu et al. (2016); Franchini, Sartor, et al. 2025; Franchini, Švajda, et al. 2025
Education level (years in school)	+	Higher education is often associated with a positive attitude toward carnivores.	Smith et al. (2014); Bhatia et al. (2017); Franchini, Sartor, et al. 2025; Franchini, Švajda, et al. 2025
Total small‐sized livestock per family	−	The preferred weight range for the snow leopard's prey varies between 36 to 76 kg. However, they are also capable of catching larger prey.	Lovari et al. (2013); Augugliaro et al. (2020)
Total large‐sized livestock per family	−		
Number of livestock guardian dogs	−	Using prevention measures may contribute to reducing livestock losses and thereby influence herders' attitudes toward carnivores. However, adoption of such measures may also be more common among herders experiencing higher levels of conflict, making the relationship between prevention strategies and tolerance potentially complex.	Augugliaro et al. (2020); Filla et al. (2022); Franchini and Guerisoli (2023)

Before being included in the model, covariates were standardized using the *mutate_at* function implemented in the ‘dplyr’ R package (Wickham et al. 2026). Multicollinearity among covariates/predictors was tested using the Variance Inflation Factor (VIF) (Fox and Monette 1992) implemented in the ‘usdm’ R package (Naimi et al. 2014). We considered VIF ≥ 3 (Hair 2014) as a threshold value to define high correlation among covariates/predictors.

#### 2.3.2 Livestock Depredations and Prey Preference Analyses in both the Pre‐Project and Project Periods

Using the either the Chi‐square test (Fisher 1922) or the one‐proportion z‐test through either the *binom.test* function (when the sample size was ≤ 30) or the *prop. test* function (when the sample size was > 30) (Agresti 2013), we analyzed the main causes of livestock losses (i.e., depredation, other mortality causes such as disease, malnutrition, severe weather, etc.), the time of the day in which the attacks and depredations occurred (i.e., day vs. night), and the differences with respect to attacks and depredations by solitary wolves, pairs and/or packs. In the analysis, we referred to ‘carnivore attacks’ to indicate the number of attacks on livestock (i.e., depredation events), and ‘carnivore depredations’ to indicate the number of depredated individuals. This distinction allows separating predation events from the number of livestock individuals affected per event. For post‐hoc pairwise comparisons between groups, the *pairwise comparison of proportions with Holm correction* (*pcp*) (Agresti 2013) was used. Because data referring to the pre‐project period are less detailed with respect to (i) the main causes of livestock losses, and (ii) the difference in terms of number of livestock attacks and depredations by solitary wolves, pairs and/or packs, these data were analyzed for the project period only.

With regards to prey selection by both snow leopard and wolf, potentially significant differences in livestock depredation rates between livestock species (i.e., sheep and goat, cattle, yak, horse) were first assessed using the Fisher's exact test (Fisher 1922). Subsequently, prey selection analysis was conducted using the Jacobs' selectivity index (Jacobs 1974). This index ranges from −1 (indicating complete avoidance) to +1 (indicating exclusive use), with preference or underuse declared only at values > 0.3 or < −0.3, respectively (Laurenzi et al. 2016; Lovari et al. 2017; Rasphone et al. 2022; Franchini et al. 2024, 2026).

#### 2.3.3 Foxlights' Effectiveness in Deterring Predators

To test the effectiveness of Foxlights as deterrents against snow leopards and wolves, the analysis was divided into two Bayesian generalized linear mixed models (BGLMMs) with a *negative binomial* distribution to account for overdispersion in the data, and one Bayesian generalized linear model (BGLM) with a *zero‐inflated negative binomial* distribution to account for both excess zeros and overdispersion (Zuur et al. 2009). All models were fitted using the ‘brms’ R package (Bürkner 2021), which is particularly suitable for small sample sizes and improves parameter estimation stability (e.g., Filacorda et al. 2022; Franchini et al. 2022; Gelman and Hill 2006; Gelman et al. 2025; Bikembo et al. 2026).

In the first BGLMM, we compared the same herders (marked with an ID) who suffered night‐time depredations on sheep and goats in the pre‐project and project periods and were not equipped with Foxlights. Conversely, in the second BGLMM, we compared the same herders who suffered night‐time depredations on sheep and goats in the pre‐project and project periods but were instead equipped with Foxlights. In both models, the total number of depredated livestock was used as the response variable, while the number of small livestock and the number of LGDs were included as fixed effects, with herder ID included as a random effect. In the second BGLMM, Foxlights presence/absence was additionally included as a fixed effect to explicitly test its effectiveness in reducing depredation events.

The BGLM was used to compare total night‐time depredation events between ‘treatment’ and ‘control’ groups in the project period. In this model, the number of depredated individuals was included as the response variable, and Foxlights presence/absence as the sole predictor. The initial aim was to include a more complex model structure including small livestock numbers, number of LGDs, and Foxlights presence/absence as fixed effects, while herder ID as a random effect. However, given the low sample size and high number of zeros, more complex specifications did not improve convergence diagnostics nor ecological interpretability, and resulted in unstable parameter estimates due to weak identifiability. Therefore, final inference was based on the hierarchical negative binomial model as the most parsimonious and ecologically coherent representation of the data‐generating process.

Effects were interpreted based on the posterior distribution of parameters, with a 95% credible interval excluding zero considered as evidence of a meaningful effect (Gelman and Hill 2006; Gelman et al. 2025). All models were run with 12,000 iterations, four chains, and 2000 warm‐up iterations. Convergence was assessed using the Rhat statistic, a standard diagnostic for MCMC convergence (Gelman et al. 2004; Kruschke 2014), with values equal to 1 indicating satisfactory convergence. Model fit was further evaluated using normal Q‐Q plots of residuals and trace plots of posterior chains (Figures S3–S8; Supporting Information S3), implemented via the ‘bayesplot’ R package (Gabry and Mahr 2024).

Prior to modeling, multicollinearity among predictors was assessed using the VIF in the ‘car’ R package (Fox and Weisberg 2019), with VIF ≥ 3 indicating high collinearity (Hair 2014). Finally, all covariates were standardized using the *mutate_at* function from the ‘dplyr’ R package (Wickham et al. 2026) to improve model convergence and parameter estimation.

## 3 Results

### 3.1 Households' Characteristics and Herders' Attitude toward the Snow Leopard and Wolf in the Pre‐Project Period

Overall, *n* = 58 herders were interviewed (*n* = 20 in Chigertei, *n* = 20 in Khokh Serkh, and *n* = 18 in Tolbo), including one respondent who also worked as a ranger. These herders collectively owned *n* = 20,562 livestock: *n* = 18,767 (91.3%) sheep and goats, *n* = 1111 (5.4%) yaks, *n* = 658 (3.2%) horses, and *n* = 26 (0.1%) cattle. The characteristics of the respondents are reported in Table 1.

All respondents showed a negative attitude toward the wolf. Conversely, for snow leopard, *n* = 24 respondents (41.4%) showed a positive attitude, another *n* = 14 (24.1%) were neutral, while *n* = 20 (34.5%) displayed a negative attitude. From the implementation of the BCMM, no clear directional effect was observed with regards to the considered covariates/predictors, that is, ‘age’ (*β* = −0.3, SE = 0.4, 95% CI = −1.0 to 0.4), ‘education level’ (*β* = 0.02, SE = 0.3, 95% CI = −0.6 to 0.6), ‘number of small livestock bred per family’ (*β* = −0.5, SE = 0.4, 95% CI = −1.2 to 0.2), ‘number of large livestock bred per family’ (*β* = 0.3, SE = 0.4, 95% CI = −0.5 to 1.1), and ‘number of LGDs’ (*β* = −0.03, SE = 0.3, 95% CI = −0.6 to 0.5).

### 3.2 Effect of Carnivores on Livestock Losses

#### 3.2.1 Characterization of both Day‐time and Night‐time Depredation Events by Snow Leopard and Wolf in both the Pre‐Project and Project Periods

The overall number of attacks and depredations on livestock by snow leopards and wolves, categorized by species and age class, during both day‐time and night‐time periods, is summarized in Table 3.

**TABLE 3:** Number of attacks and depredated livestock individuals by snow leopards and wolves (both during day and night), divided by species and age class, within each area.

Locality	Project period	Livestock	Age class	Snow leopard				Wolf			
				Attacks		Depredations		Attacks		Depredations	
				*n*	%	*n*	%	*n*	%	*n*	%
CH	—	Sheep/goat	Adult	3	42.9	4	44.4	24	30.0	60	46.9
			Young/sub‐adult	0	0	0	0	26	32.5	32	25
			NA	0	0	0	0	1	1.3	2	1.6
		Yak	Adult	0	0	0	0	0	0	0	0
			Young/subadult	3	43	4	44	14	17.5	18	14.1
			NA	0	0	0	0	1	1.3	2	1.6
		Horse	Adult	0	0	0	0	1	1.3	1	0.8
			Young/sub‐adult	1	14	1	11	13	16.3	13	10.2
			NA	0	0	0	0	0	0	0	0
	**Total**			**7**	**100**	**9**	**100**	**80**	**100**	**128**	**100**
KS	Pre	Sheep/goat	Adult	13	56.5	32	72.7	17	51.5	152	83.1
			Young/sub‐adult	0	0	0	0	7	21.2	18	9.8
			NA	0	0	0	0	0	0	0	0
		Yak	Adult	1	4.3	1	2.3	2	6.1	4	2.2
			Young/subadult	8	34.8	10	22.7	2	6.1	3	1.6
			NA	0	0	0	0	0	0	0	0
		Horse	Adult	1	4.3	1	2.3	2	6.1	3	1.6
			Young/sub‐adult	0	0	0	0	3	9.1	3	1.6
			NA	0	0	0	0	0	0	0	0
	**Total**			**23**	**100**	**44**	**100**	**33**	**100**	**183**	**100**
	During	Sheep/goat	Adult	1	2.9	2	4.4	16	32.7	21	24.7
			Young/sub‐adult	0	0	0	0	15	30.6	28	32.9
			NA	4	11.4	8	17.8	1	2	3	3.5
		Yak	Adult	0	0	0	0	0	0	0	0
			Young/sub‐adult	20	57.1	22	48.9	4	8.2	7	8.2
			NA	0	0	0	0	0	0	0	0
		Horse	Adult	0	0	0	0	0	0	0	0
			Young/sub‐adult	9	25.7	11	24.4	10	20.4	20	23.5
			NA	1	2.9	2	4.4	3	6.1	6	7.1
	**Total**			**35**	**100**	**45**	**100**	**49**	**100**	**85**	**100**
TB	Pre	Sheep/goat	Adult	1	14.3	2	15.4	14	43.8	149	72.7
			Young/subadult	0	0	0	0	3	9.4	9	4.4
			NA	0	0	0	0	0	0	0	0
		Yak	Adult	3	42.9	5	38.5	4	12.5	9	4.4
			Young/subadult	0	0	0	0	1	3.1	2	1
			NA	0	0	0	0	0	0	0	0
		Horse	Adult	2	28.6	5	38.5	7	21.9	29	14.1
			Young/subadult	1	14.3	1	7.7	3	9.4	7	3.4
			NA	0	0	0	0	0	0	0	0
	**Total**			**7**	**100**	**13**	**100**	**32**	**100**	**205**	**100**
	During	Sheep/goat	Adult	1	100	1	100	20	25.6	32	31.4
			Young/sub‐adult	0	0	0	0	29	37.2	39	38.2
			NA	0	0	0	0	0	0	0	0
		Yak	Adult	0	0	0	0	0	0	0	0
			Young/sub‐adult	0	0	0	0	15	19.2	15	14.7
			NA	0	0	0	0	0	0	0	0
		Horse	Adult	0	0	0	0	0	0	0	0
			Young/sub‐adult	0	0	0	0	14	17.9	16	15.7
			NA	0	0	0	0	0	0	0	0
	**Total**			**1**	**100**	**1**	**100**	**78**	**100**	**102**	**100**

*Note:* Period subdivision, that is, pre‐project vs. project periods, was provided only for Tolbo (TB) and Khokh Serkh (KS) as no Foxlights were distributed in Chigertei (CH). NA (not available) was used for those cases in which the age class was not identified.

The differentiation between pre‐project and project periods was provided for Tolbo and Khokh Serkh only, as no Foxlights were distributed in Chigertei (Table 3). Most livestock attacks and depredation events by snow leopards and wolves on sheep and goats involved adult and sub‐adult/young individuals. In contrast, the majority of attacks and depredation events involving yaks and horses affected sub‐adults and young animals (Table 3).

In the pre‐project period, livestock attacks by snow leopards were significantly higher (*one‐proportion z‐test*, *p* < 0.001) during the day (*n* = 25, 83.3%) compared to night (*n* = 5, 16.7%). A similar significant trend (*one‐proportion z‐test*, *p* < 0.001) was observed for livestock depredations, with *n* = 49 (86%) depredations occurred during the day and *n* = 8 (14%) in the night, accounting for 0.4% of the total livestock owned. Similarly, livestock attacks by wolves were significantly higher (*one‐proportion z‐test*, *p* < 0.001) during the day (*n* = 49, 75.4%) compared to night (*n* = 16, 24.6%). This significant trend (*one‐proportion z‐test*, *p* < 0.001) was also reflected in depredations, with *n* = 253 (65.2%) depredations registered during the day and *n* = 135 (34.8%) in the night, corresponding to 2.9% of the total livestock owned.

In the project period, all livestock attacks by snow leopard occurred during the day (*n* = 36) resulting in *n* = 46 depredated individuals, accounting for 0.3% of the total livestock owned. No attacks/depredations at night were registered. As for the wolf, livestock attacks were significantly higher (*one‐proportion z‐test*, *p* < 0.001) during the day (*n* = 122, 96.1%) compared to night (*n* = 5, 3.9%). Similarly, a significant trend (*one‐proportion z‐test*, *p* < 0.001) was observed for livestock depredations, with *n* = 145 (77.5%) depredations recorded during the day and *n* = 42 (22.5%) at night, corresponding to 1.4% of the total livestock owned.

**FIGURE 3:**
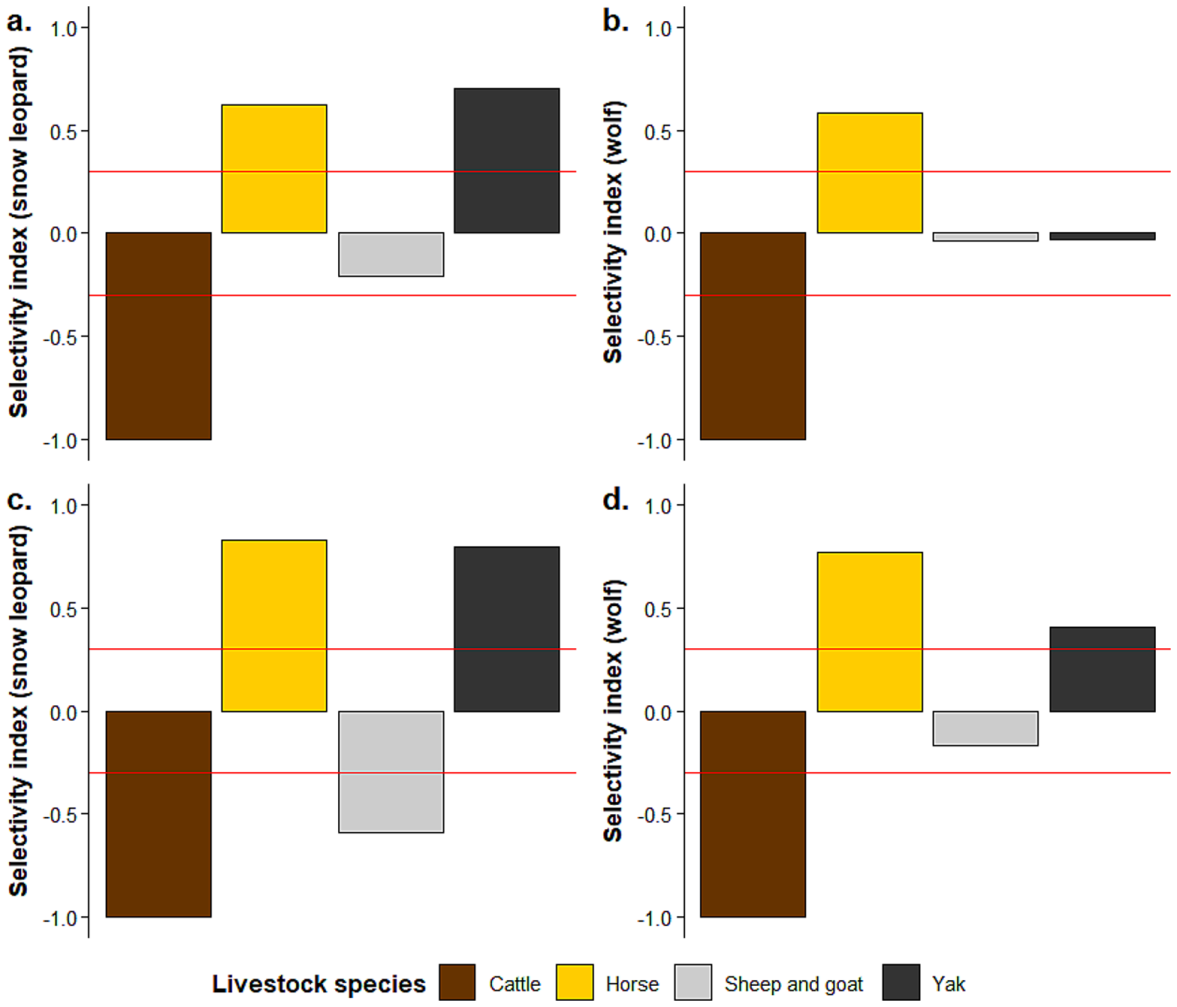
Prey selection (Jacobs' selectivity index) exhibited by (a, c) snow leopard and (b, d) wolf toward each livestock species (cattle, horse, sheep and goat, yak), during both day and night, in the pre‐project period (a, b) as well as in the project period (c, d), in the localities of Tolbo and Khokh Serkh only. Horizontal red lines indicate a preference (> 0.3) or underuse (< −0.3) toward a certain species.

In the pre‐project period, for both the snow leopard and wolf, livestock depredations (both day and night) differed significantly from expected values (Fisher's test, *p* < 0.001). Specifically, the snow leopard exhibited a preference for both horses (*D* = 0.6) and yaks (*D* = 0.7), while sheep and goats were preyed upon according to their availability (*D* = −0.2). A complete avoidance was shown for cattle (*D* = −1) (Figure 3a). Similarly, in the pre‐project period, the wolf displayed a preference for horses (*D* = 0.5), while yaks (*D* = −0.03) and sheep and goats (*D* = −0.04) were preyed upon according to their availability. A complete avoidance was also shown for cattle (*D* = −1) (Figure 3b).

In the project period, for both snow leopard and wolf livestock depredation patterns (both day and night) differed significantly from expected values (Fisher's test, *p* < 0.001). Specifically, the snow leopard appeared to exhibit a preference for horses (*D* = 0.8) and yaks (*D* = 0.8), while sheep and goats were underrepresented (*D* = −0.6) in stock lost from depredation. A complete avoidance was shown for cattle (*D* = −1) (Figure 3c). Similarly, in the project period, the wolf displayed a preference for horses (*D* = 0.8) and yaks (*D* = 0.4), while sheep and goats were preyed upon according to their availability (*D* = −0.2). A complete avoidance was shown for cattle (*D* = −1) (Figure 3d).

#### 3.2.2 Foxlights' Effectiveness in Reducing Night‐time Livestock Depredations by Snow Leopard and Wolf

In the pre‐project period, *n* = 17 night‐time attacks on sheep and goats were recorded, including *n* = 1 (5.9%) attributed to snow leopards and *n* = 16 (94.1%) to wolves. These attacks resulted in the depredation of *n* = 3 (2.2%) livestock individuals by snow leopards and *n* = 135 (97.8%) by wolves. In the project period, the number of night‐time attacks on sheep and goats decreased to *n* = 5, all attributed to wolves, resulting in *n* = 42 depredated individuals (Table 4).

**TABLE 4:** Number of attacks and depredated sheep and goat individuals during the night by snow leopards and wolves in the pre‐project and project periods, with households selected as ‘treatment’ groups reported in *italics*.

ID	Locality	Small livestock number	LGDs	Pre‐project period				Project period			
				Foxlights	Responsible predator	Attack	Depredations	Foxlights	Responsible predator	Attack	Depredations
*10KS90*	*KS*	*253*	*0*	*No*	—	*0*	*0*	*Yes*	—	*0*	*0*
10TL90	TB	107	0	No	—	0	0	No	—	0	0
*11KS90*	*KS*	*96*	*1*	*No*	*Wolf*	*1*	*11*	*Yes*	—	*0*	*0*
*11TL90*	*TB*	*763*	*1*	*No*	*Wolf*	*1*	*6*	*Yes*	*Wolf*	*1*	*6*
12KS90	KS	417	0	No	—	0	0	No	—	0	0
12TL90	TB	273	1	No	Wolf	1	11	No	Wolf	1	11
13KS90	KS	479	1	No	—	0	0	No	—	0	0
13TL90	TB	433	0	No	Wolf	1	16	No	Wolf	1	16
*14KS90*	*KS*	*393*	*1*	*No*	—	*0*	*0*	*Yes*	—	*0*	*0*
*14TL90*	*TB*	*197*	*1*	*No*	*Wolf*	*1*	*5*	*Yes*	*Wolf*	*1*	*5*
*15KS90*	*KS*	*333*	*1*	*No*	—	*0*	*0*	*Yes*	—	*0*	*0*
15TL90	TB	121	1	No	Wolf	1	4	No	Wolf	1	4
*16KS90*	*KS*	*413*	*0*	*No*	—	*0*	*0*	*Yes*	—	*0*	*0*
16TL90	TB	337	2	No	—	0	0	No	—	0	0
17KS90	KS	99	1	No	Snow leopard	1	3	No	—	0	0
17TL90	TB	119	0	No	—	0	0	No	—	0	0
*18KS90*	*KS*	*213*	*1*	*No*	*Wolf*	*1*	*6*	*Yes*	—	*0*	*0*
18TL90	TB	583	1	No	—	0	0	No	—	0	0
19KS90	KS	652	1	No	—	0	0	No	—	0	0
*1KS90*	*KS*	*381*	*0*	*No*	*Wolf*	*1*	*6*	*Yes*	—	*0*	*0*
*1TL90*	*TB*	*376*	*2*	*No*	—	*0*	*0*	*Yes*	—	*0*	*0*
*20KS90*	*KS*	*144*	*0*	*No*	—	*0*	*0*	*Yes*	—	*0*	*0*
*2KS90*	*KS*	*397*	*0*	*No*	*Wolf*	*1*	*11*	*Yes*	—	*0*	*0*
*2TL90*	*TB*	*467*	*2*	*No*	*Wolf*	*1*	*6*	*Yes*	—	*0*	*0*
3KS90	KS	439	1	No	Wolf	1	17	No	—	0	0
*3TL90*	*TB*	*300*	*2*	*No*	*Wolf*	*1*	*9*	*Yes*	—	*0*	*0*
4KS90	KS	308	0	No	Wolf	1	5	No	—	0	0
*4TL90*	*TB*	*305*	*1*	*No*	*Wolf*	*1*	*12*	*Yes*	—	*0*	*0*
5KS90	KS	395	1	No	—	0	0	No	—	0	0
*5TL90*	*TB*	*92*	*0*	*No*	—	*0*	*0*	*Yes*	—	*0*	*0*
6KS90	KS	593	0	No	Wolf	1	4	No	—	0	0
6TL90	TB	452	1	No	—	0	0	No	—	0	0
*7KS90*	*KS*	*303*	*1*	*No*	—	*0*	*0*	*Yes*	—	*0*	*0*
*7TL90*	*TB*	*236*	*1*	*No*	—	*0*	*0*	*Yes*	—	*0*	*0*
*8KS90*	*KS*	*203*	*0*	*No*	—	*0*	*0*	*Yes*	—	*0*	*0*
8TL90	TB	164	1	No	Wolf	1	6	No	—	0	0
*9KS90*	*KS*	*323*	*1*	*No*	—	*0*	*0*	*Yes*	—	*0*	*0*
9TL90	TB	182	1	No	—	0	0	No	—	0	0
**Total**	**—**	**12,341**	**29**	**—**	**—**	**17**	**138**	**—**	**—**	**5**	**42**

*Note:* ‘Small livestock number’ indicates the number of small‐sized livestock bred by each herder. Abbreviations: LGDs = number of livestock guardian dog(s), KS = Khokh Serkh, TB = Tolbo.

**FIGURE 4:**
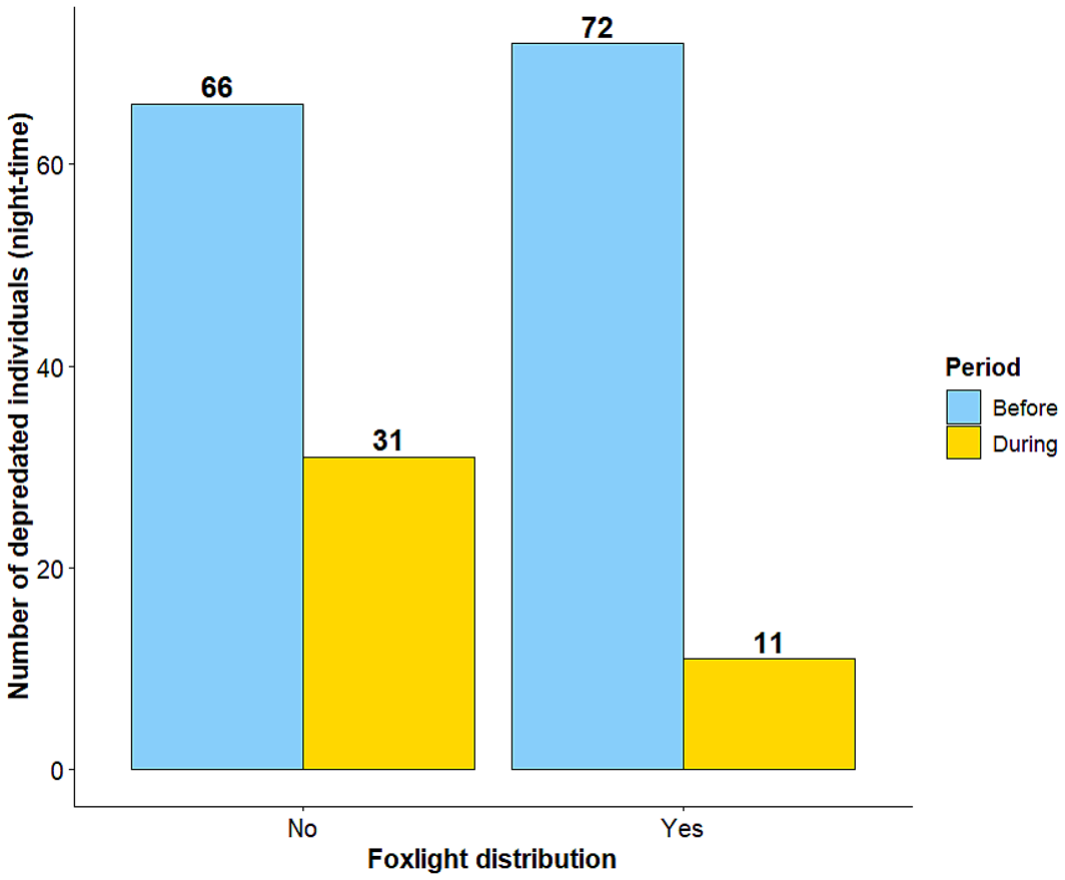
Effectiveness of Foxlights in reducing night‐time depredation events on sheep and goats, in the localities of Tolbo and Khokh Serkh only. The chart shows depredation events experienced by the same herders by comparing the pre‐project and project periods, as well as between ‘treatment’ and ‘control’ groups in the project period only.

From the comparison of night‐time depredation events on sheep and goats among the same herders not equipped with Foxlights across the pre‐project and project periods, results showed that *n* = 66 depredation events were recorded in the pre‐project period, whereas *n* = 31 events were observed in the project period (Figure 4). The BGLMM revealed no strong directional effects for the fixed factors ‘number of small livestock’ (*β* = −0.8, SE = 1.2, 95% CI = −3.3 to 1.4) and ‘number of LGDs’ (*β* = −0.8, SE = 1.2, 95% CI = −3.4 to 1.4). Conversely, from the comparison of night‐time depredation events on sheep and goats across the pre‐project and project periods among the same herders equipped with Foxlights, *n* = 72 depredation events were recorded before project implementation, whereas only *n* = 11 were observed afterwards (Figure 4). The corresponding BGLMM revealed no strong directional effects for the fixed factors ‘number of small livestock’ (*β* = 0.8, SE = 0.9, 95% CI = −0.7 to 2.9) and ‘number of LGDs’ (*β* = 0.8, SE = 1.0, 95% CI = −1.0 to 3.0). In contrast, a clear directional effect was observed for the fixed factor ‘presence/absence of Foxlights’ (*β* = −3.6, SE = 1.4, 95% CI = −6.9 – −1.2). Specifically, Foxlights were associated with a substantial reduction in depredation risk (e^*β*^), corresponding to an approximate 30‐fold decrease compared to the pre‐project period.

As for the comparison between ‘treatment’ and ‘control’ groups in the project period only, among the *n* = 20 ‘treatment’ sites, *n* = 2 (10.0%) experienced livestock attacks, resulting in *n* = 11 depredated individuals. In contrast, among the *n* = 18 ‘control’ sites, *n* = 3 (16.7%) experienced attacks, with a total of *n* = 31 depredated individuals (Figure 4). The BGLM revealed a clear directional effect of the predictor ‘presence/absence of Foxlights’ (*β* = 1.0, SE = 0.1, 95% CI = 0.9–1.2). Specifically, the risk of depredation was approximately 2.7 times higher (e^*β*^) in ‘control’ groups compared to ‘treatment’ groups equipped with Foxlights.

#### 3.2.3 Main Causes of Livestock Losses in the Project Period

**FIGURE 5:**
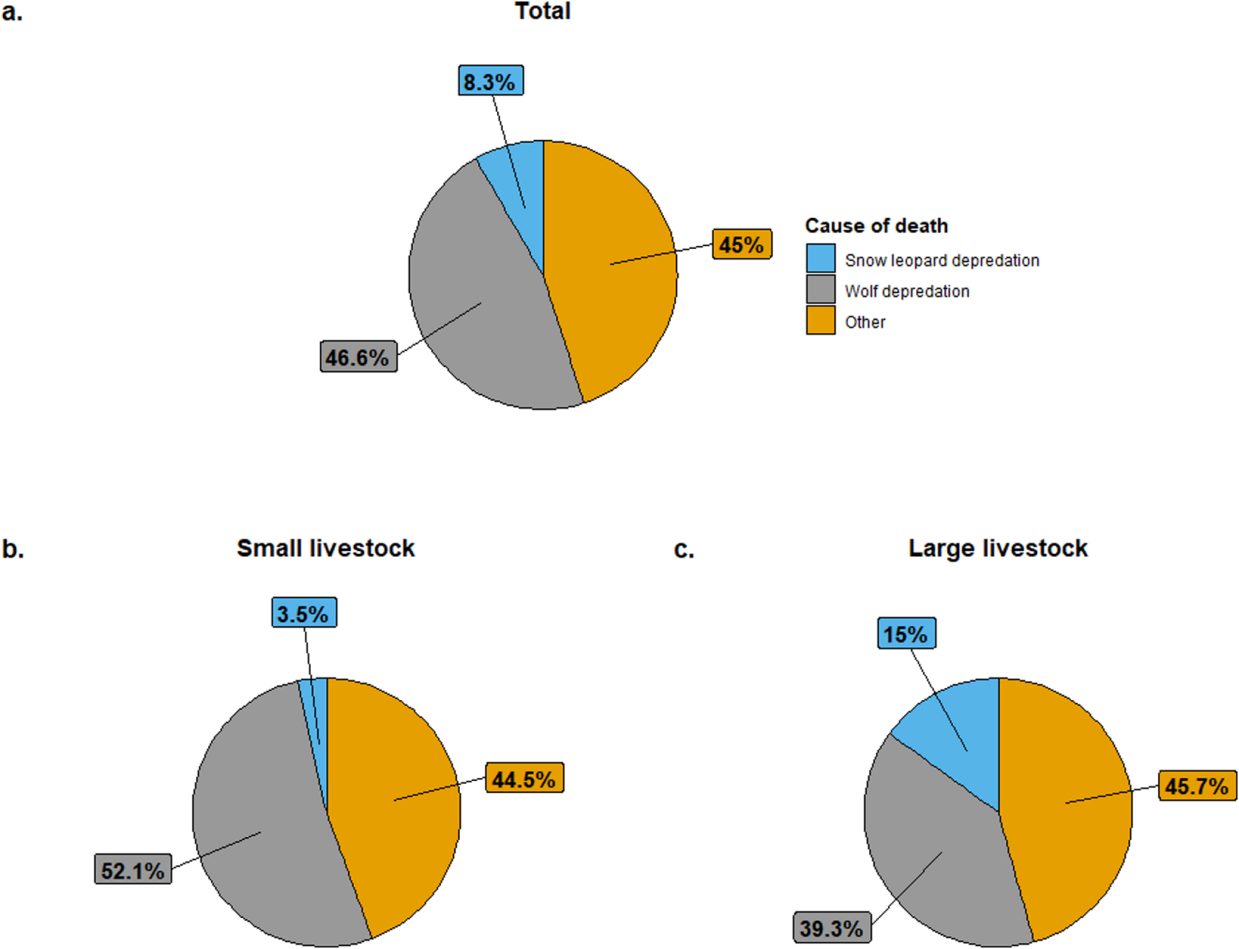
Main causes of total (small livestock—sheep and goat, large livestock—cattle, yak, horse) (a), small (b), and large livestock losses (c) collected from 58 households in the Bayan‐Ölgii province, in the project period. Livestock losses due to carnivore attacks include both day and night depredation events. The category ‘other’ refers to nonpredatory factors such as disease, malnutrition, severe weather, climate, etc.

Most small and large livestock losses were attributed to wolf depredation (*n* = 257, 46.6%) closely followed by other mortality sources (e.g., disease, malnutrition, severe weather, climate) (*n* = 248, 45.0%) and lastly attributed to snow leopard depredation (*n* = 46, 8.3%) (Figure 5a). A significant difference (χ^2^ = 155.0, *p* < 0.001) was found between categories. Specifically, the total livestock losses due to wolf depredation and other causes were significantly higher (*pcp*, *p* < 0.001) than the total livestock losses due to snow leopard depredation (Figure 5a).

Most small‐bodied livestock losses are associated with wolf depredation (*n* = 165, 52.1%), followed by other causes (*n* = 141, 44.5%) and lastly snow leopard depredation (*n* = 11, 3.5%) (Figure 5b). A significant difference (χ^2^ = 129.9, *p* < 0.001) was found between categories, with small livestock losses due to both wolf depredation and other causes being significantly higher (*pcp*, *p* < 0.001) than losses attributed to snow leopard (Figure 5b).

Most large livestock loss is associated with other causes (*n* = 107, 45.7%), followed by wolf (*n* = 92, 39.3%) and snow leopard depredation (*n* = 35, 15.0%), respectively (Figure 5c). A significant difference (χ^2^ = 37.0, *p* < 0.001) was found between categories, with large livestock losses due to both wolf depredation and other causes being significantly higher (*pcp*, *p* < 0.001) compared to snow leopard depredation (Figure 5c).

#### 3.2.4 Impact of Solitary Wolves, Pairs and Packs on Livestock in the Project Period

The number of wolf attacks and depredations (solitary individuals vs. pairs vs. packs) on livestock in each area (Chigertei, Tolbo and Khokh Serkh) according to species and age class are summarized in Table 5.

**TABLE 5:** Number of livestock attacks and depredations (both day and night) by wolves (solitary individuals vs. pairs vs. packs) by species and age class, in the project period and across all localities (Chigertei, Tolbo, and Khokh Serkh).

Livestock	Age class	Solitary				Pairs				Packs				NA			
		Attacks		Depredations		Attacks		Depredations		Attacks		Depredations		Attacks		Depredations	
		*n*	%	*n*	%	*n*	%	*n*	%	*n*	%	*n*	%	*n*	%	*n*	%
Sheep/goat	Adult	34	27.0	42	26.8	6	17.6	7	16.3	6	27.3	13	26.5	2	50.0	6	75.0
	Young/sub‐adult	50	39.7	69	43.9	14	41.2	19	44.2	2	9.1	3	6.1	1	25.0	1	12.5
	NA	1	0.8	3	1.9	0	0.0	0	0.0	1	4.5	2	4.1	0	0.0	0	0.0
Yak	Adult	0	0.0	0	0.0	0	0.0	0	0.0	0	0.0	0	0.0	0	0.0	0	0.0
	Young/sub‐adult	21	16.7	21	13.4	7	20.6	10	23.3	3	13.6	7	14.3	0	0.0	0	0.0
	NA	0	0.0	0	0.0	0	0.0	0	0.0	1	4.5	2	4.1	0	0.0	0	0.0
Horse	Adult	0	0.0	0	0.0	0	0.0	0	0.0	0	0.0	0	0.0	0	0.0	0	0.0
	Young/sub‐adult	20	15.9	22	14.0	7	20.6	7	16.3	6	27.3	16	32.7	1	25.0	1	12.5
	NA	0	0.0	0	0.0	0	0.0	0	0.0	3	13.6	6	12.2	0	0.0	0	0.0
**Total**		**126**	**100**	**157**	**100**	**34**	**100**	**43**	**100**	**22**	**100**	**49**	**100**	**4**	**100**	**8**	**100**

Abbreviation: NA = not available data.

**FIGURE 6:**
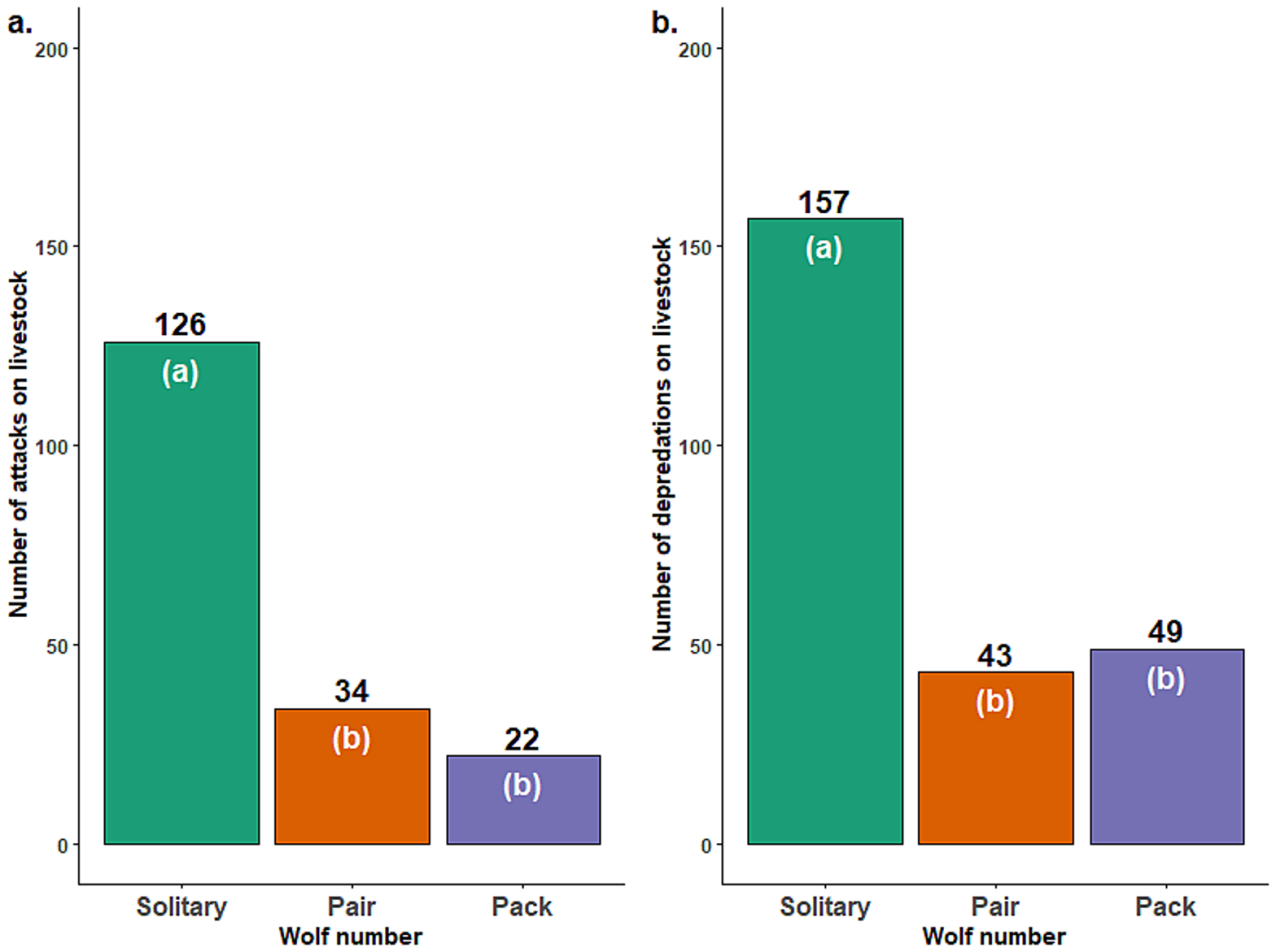
Impact of solitary wolves, pairs and/or packs in terms of livestock attacks (a) and depredations (b), during both day and night, in the project period across all localities (Chigertei, Tolbo, and Khokh Serkh). Different letters indicate statistically significant differences between bars based on post‐hoc comparisons.

The number of wolves involved in livestock attacks differed significantly (χ^2^ = 106.7, *p* < 0.001) comparing solitary individuals, pairs, or packs. Specifically, the attacks by solitary individuals were significantly higher (*pcp*, *p* < 0.001) than those by pairs and/or packs (Figure 6a). A similar significant pattern (χ^2^ = 99.2, *p* < 0.001) was observed with regards to the number of wolves involved in livestock depredations. Namely, the number of depredations by solitary individuals was significantly higher (*pcp*, *p* < 0.001) than the one by pairs and/or packs (Figure 6b).

## 4 Discussion

This study marks the first direct involvement of Mongolian herders in field data collection, demonstrating the effectiveness of this approach in generating high‐resolution, weekly data on livestock depredation rates. The information gathered may aid in implementing management and conservation strategies to promote human–carnivore coexistence in shared landscapes. Additionally, the use of Foxlights has high potential in reducing night‐time depredations on livestock kept in corrals or close to the campsite, thus enhancing conservation efforts.

### 4.1 Herder Attitudes and Carnivores' Impact on Livestock Activities

None of the covariates/predictors considered in the analysis (i.e., age, education level, number of small and large livestock per family, and number of LGDs) explained attitudes toward the snow leopard. However, in line with our initial hypothesis, we observed a consistently negative attitude toward the wolf and a comparatively more positive one toward the snow leopard. Compared to snow leopards, wolves are responsible of the majority of livestock attacks and depredations (Mijiddorj et al. 2018; Augugliaro et al. 2020) in turn driving a more negative attitude toward the species. However, unmeasured factors, such as cultural traditions and/or religious beliefs, may also explain the differences in tolerance toward these two carnivores. In areas where herders share the landscapes with more carnivore species, wolves often receive the most negative attitudes (e.g., Alexander et al. 2015; Dressel et al. 2015; Augugliaro et al. 2020). Across Asia, wolves are often hunted due to their real or perceived impact on livestock, long‐standing folklore and cultural narratives, whereas big cats are more charismatic and culturally valued (Werhahn et al. 2025). In Mongolia, prior to the establishment of Socialism in 1924, wolves were regarded with respect. However, in contemporary Mongolian society, they are heavily persecuted and are subject to intense wildlife trade involving wolf parts (Werhahn et al. 2025). In most cases, the wolf is considered responsible for the majority of attacks on both small and large livestock, with all herders having negative attitudes toward this species (Augugliaro et al. 2020). Conversely, according to other research conducted in Nepal (Kusi et al. 2019), China (Li, Yin, et al. 2013; Alexander et al. 2015), India (Suryawanshi et al. 2013), and Mongolia (Augugliaro et al. 2020), and despite some records of attacks on livestock, local inhabitants generally show positive attitudes toward the snow leopard. Snow leopards have higher cultural and religious status compared to wolves (Kusi et al. 2019), and are frequently perceived as mystical and revered animals, particularly within Buddhist communities (Li, Wang, et al. 2013). Consequently, attitudes toward snow leopards are generally more positive than those toward wolves.

Considering the total livestock owned by the interviewed herders, the percentage of livestock lost to both day‐time and night‐time depredation events during both the pre‐project and project periods was 0.7% and 4.3% for snow leopards and wolves, respectively. These findings support our initial hypothesis regarding the greater impact that wolves may have on livestock. For the snow leopard, this value falls within the range observed in other studies (Li, Yin, et al. 2013; Alexander et al. 2015; Augugliaro et al. 2020). Conversely, the impact of the wolf was six times higher, aligning more closely with the results reported in China by Alexander et al. (2015) (3X greater) and Li, Yin, et al. (2013) (4X greater), but considerably lower compared to those reported in western Mongolia by Augugliaro et al. (2020) (20X greater). However, both research projects by Li, Yin, et al. (2013) and Augugliaro et al. (2020) were conducted on a larger spatial scale and involved more households, hence making results hard to compare.

According to other studies (e.g., Guerisoli et al. 2017; Janeiro‐Otero et al. 2020; Franchini and Guerisoli 2023; Franchini, Sartor, et al. 2025; Franchini, Raniolo, et al. 2025; Frangini et al. 2025), sheep and goats turned out to be the most affected livestock category by carnivores likely because of their smaller size, absence of defensive weaponry (e.g., robust hooves and/or horns), and/or their poor antipredatory behaviors. Furthermore, despite sheep and goats being guarded by herders and/or LGDs during the day, most herders maintain many small‐sized livestock. According to Vos (2000), larger flocks are more challenging to monitor and protect. Additionally, goats tend to graze on steep slopes and/or farther from human settlements, which complicates protection efforts and makes them more susceptible to carnivore attacks (Torres et al. 2015). Nevertheless, when considering the number of actual depredation events in relation to livestock availability, both snow leopard and wolf showed slight shifts in prey preferences in both the pre‐project and project periods. Specifically, in contrast to our initial hypothesis, the snow leopard consistently preferred unattended yaks and horses in both periods, while sheep and goats were used in proportion to their availability in the pre‐project period, but were underutilized afterward. In the case of the wolf, the species showed a preference for horses in the pre‐project period, and for both yaks and horses afterward. Sheep and goats were instead used according to their availability in both periods, again contradicting our initial expectations. While both wolves and snow leopards are capable of preying upon species weighing as much as 200 kg (Janeiro‐Otero et al. 2020; Krofel et al. 2021), the preference mostly shown for young/sub‐adult yaks and/or horses further confirm their tendency to focus their attention on more easily subdued and unattended prey (Lovari et al. 2013; Janeiro‐Otero et al. 2020). The complete avoidance shown for cattle does not align with the observations of Augugliaro et al. (2020), who noted that snow leopards also selected this livestock category. However, in our sample of interviewed herders, only four raised a small number of cattle, in turn potentially influencing the results achieved. Additionally, observed differences in livestock depredation patterns among species are likely influenced by contrasting husbandry practices, with sheep and goats benefiting from active day‐time patrolling and night‐time enclosure, whereas larger livestock are continuously free‐ranging and therefore more exposed to carnivore encounters.

### 4.2 Foxlights' Effectiveness in Reducing Night‐time Livestock Attacks and Depredations by Snow Leopard and Wolf

Our findings revealed that, in the pre‐project period, both groups of the same herders subsequently assigned to the ‘treatment’ and ‘control’ categories experienced a high number of night‐time depredation events. In the project period, the overall number of night‐time depredations decreased in both groups, but the reduction was substantially greater among herders equipped with Foxlights. Moreover, model outputs identified Foxlights' presence as the only predictor exerting a clear directional effect on depredation risk. Nevertheless, parameter estimates were associated with relatively large standard errors and wide credible intervals, suggesting some uncertainty in model estimation. This likely reflects the limited sample size and the inclusion of multiple covariates within the same modeling framework, which may have reduced parameter identifiability. However, when comparing ‘treatment’ and ‘control’ groups using a simplified model that included only Foxlights' presence/absence as a predictor, parameter estimates were more stable and confidence intervals more precise. This approach provided clearer inference and more reliable effect estimation.

Although only one snow leopard night‐time depredation event was observed in the pre‐project period, the reduction in night‐time livestock depredation by wolves, together with the absence of night‐time depredations by snow leopards following Foxlights' deployment, supports our initial hypothesis regarding the deterrent effectiveness of the device. The observed reduction in night‐time livestock depredations by wolves, together with the absence of recorded night‐time depredations by snow leopards following Foxlights implementation, is consistent with our initial hypothesis. Our findings are broadly in line with previous studies reporting reductions in livestock depredation associated with visual deterrents in different ecological contexts, including lions, pumas, and common leopards (Lesilau et al. 2018; Ohrens et al. 2019; Naha et al. 2020). However, contrasting results have also been reported, such as in the case of Andean foxes, where no clear treatment effect was detected (Ohrens et al. 2019).

In our study, Foxlights did not eliminate wolf depredation but were associated with a substantial reduction in recorded events. However, the evaluation was conducted over a single annual cycle, and longer‐term monitoring across a larger number of sites and sampling units (both ‘treatment’ and ‘control’ herders) would be required to obtain more robust and generalizable estimates of effectiveness. Finally, potential spillover effects, whereby changes in predation risk may extend beyond treated households to neighboring non‐treated ones, cannot be excluded and warrant further investigation. Such effects may arise from complex interactions between carnivore behavior and livestock management practices (Salerno et al. 2025), although they were not explicitly tested in the present study.

### 4.3 Main Causes of Livestock Losses and Impact of Solitary Wolves, Pairs and Packs on Livestock in the Project Period

The percentage of livestock losses from non‐predatory factors (45%) represents a considerable portion of the overall livestock mortality in the study area. As elsewhere in Mongolia, livestock deaths due to non‐predatory factors are frequently associated with temperature and related weather conditions imposed by harsh (Begzsuren et al. 2004) or milder (Augugliaro et al. 2020) winters. This partially aligns with findings in Nepal by Filla et al. (2022) who documented most livestock losses in Upper Mustang resulted from accidents and diseases, while a smaller percentage was associated with carnivores. Conversely, in Nepal's Manang area, depredation by carnivores (especially snow leopards) was considered the main source of livestock mortality (Filla et al. 2022).

In line with our initial hypothesis, solitary wolves were responsible for the majority of livestock attacks and depredations compared to wolf pairs and/or packs. Following optimal foraging theory, animals adopt foraging strategies that provide the highest benefits at the lowest costs to maximize fitness (Werner and Hall 1974). As suggested by our findings, this may reflect a tendency of younger solitary wolves being more inclined to attack easily catchable prey, that is, livestock (Meriggi et al. 1996; Meriggi and Lovari 1996). This assumption is also corroborated by other research (Linnell et al. 1999; Imbert et al. 2016; Mayer et al. 2022) showing that solitary dispersers in agricultural lands are more inclined to prey upon livestock.

### 4.4 Conservation and Management Recommendations

Our study relied on timely records collected daily by trained herders, supplemented by a form filled out on a weekly basis. This approach significantly enhanced the reliability and accuracy of our findings. Community‐based carnivore monitoring represents a valuable tool, as it enables herders involved in data collection to improve their ability to distinguish between depredation events caused by different carnivore species and to differentiate true depredation from scavenging behavior. Additionally, a deep knowledge and understanding of carnivore biology and their involvement in conservation initiatives may ultimately lead to more positive attitudes toward these species (Werhahn et al. 2025).

However, despite being trained, erroneous identifications might have sometimes affected herders' reports. For example, scavenging behaviors on the carcass of an animal dying from other causes can be erroneously attributed to depredation (Oli et al. 1994), especially for unguarded large‐sized livestock. Even when detailed information is collected, some depredation claims are difficult to validate (Mishra 1997). Additionally, herders' stronger negative attitude exhibited toward wolves may influence their reported depredations attributed to this species. Indeed, the way carnivores interact with herders can significantly shape their attitudes, potentially leading wolves to be considered as responsible for more damage than they actually cause (Lescureux and Linnell 2010). Lastly, although herders were trained to follow the tracks further away from a carcass to improve accuracy in identifying the number of responsible carnivores, distinguishing between two or more wolves based on footprints near a carcass might be challenging in certain contexts, in turn leading to potential erroneous identifications. In spite of these considerations, Mongolian herders have a long‐standing tradition of coexisting with carnivores and possess a remarkable ability to interpret environmental signs, especially after receiving appropriate training (Augugliaro et al. 2020). Therefore, despite such challenges, our study offers significant insights on human‐carnivore conflict in the Bayan‐Ölgii province. Additionally, although we are aware that the effectiveness of visual deterrents (and other prevention measures, more broadly) should be tested over a longer period, it provides the first important preliminary contribution to assessing the effectiveness of Foxlights in reducing night‐time carnivore depredations.

Our findings revealed that, compared to the total available livestock, impact from both snow leopards and wolves is relatively low in this study area. However, this finding needs to be interpreted with the understanding that some herders experienced higher depredation rates than others. Single‐case scenarios thus need to be considered. Despite some negative responses, the general attitude toward the snow leopard was shown to be neutral/positive. This suggests that local communities can play a pivotal role in conserving this threatened species. Conversely, urgent attention and special considerations are needed for developing conservation measures targeting wolves, as all respondents held negative attitudes toward this animal. Foxlights proved effective in deterring night‐time carnivore attacks, with number of livestock depredations consistently reduced based on a comparison between ‘treatment’ and ‘control’ groups, as well as between pre‐project and project periods. Although visual deterrents are known for their temporal effectiveness (Eklund et al. 2017), the nomadic behavior of herders in Mongolia helps slow the carnivores' habituation process. In these areas, herders relocate up to 5 km each season, and Foxlights are reinstalled after each movement. Although both wolves and snow leopards have large home‐ranges (Mattisson et al. 2013 for wolves; Johansson et al. 2018 for snow leopards), relocating Foxlights along the perimeter of each newly erected stone corral or within the campsite (in the few cases where sheep and goats are not enclosed in corrals at night but kept near the campsite) may represent a novel stimulus for carnivores. Nevertheless, none of the attacks at the ‘treatment’ sites occurred in areas where sheep and goats were not enclosed within corrals during the night. Therefore, it is possible that the observed depredations are also linked to the improper placement of Foxlights along corral perimeters. For effectiveness, Foxlights must be carefully and properly set up. This assumes particular importance in late winter, coinciding with the wolf dispersal period (Jimenez et al. 2017; Morales‐González et al. 2022), when solitary and/or young individuals may relay on the most easily catchable prey such as livestock (Linnell et al. 1999; Imbert et al. 2016; Mayer et al. 2022). Therefore, increasing knowledge and understanding among herders regarding optimal placement of these devices and raising awareness for the timely implementation of prevention measures, strengthens their sense of ownership, in turn leading to more positive results (Eklund et al. 2017).

In the pre‐project period, LGDs were already in use in 65% of the treatment sites and in approximately 67% of the control sites. However, the results obtained from the model revealed that the presence or absence of Foxlights was the only factor significantly contributing to reduce night‐time depredation events. However, despite this encouraging result, some night depredations by wolves at the expense of sheep and goats still occurred. The effectiveness of non‐lethal interventions varies depending on several factors, such as local husbandry practices, environmental/ecological conditions, and social systems. Therefore, case‐specific scenarios should be considered and applied. These findings highlight the importance of considering system complexity when evaluating prevention strategies. In our study area, carnivore behavior, livestock composition, herding practices, LGDs' presence, and spatial configuration of households interact within a socio‐ecological system. The observed outcomes are therefore unlikely to reflect a simple treatment effect, but rather the interaction between deterrent devices and this broader ecological and social context. Long‐term studies incorporating a broader range of environmental, ecological, social, and livestock management factors are therefore needed to provide more comprehensive insights into the drivers of livestock depredations and the effectiveness of implemented preventive measures.

Although achievable in certain contexts (Carter et al. 2012; Schuette et al. 2013), human‐carnivore coexistence still represents a major global challenge (Woodroffe et al. 2005; Treves et al. 2006; Dickman et al. 2011). By coexistence, we refer to a sustainable yet dynamic state in which humans and wildlife coadapt to shared landscapes, and where human‐wildlife interactions are effectively governed to ensure the persistence of wildlife populations in socially legitimate ways that maintain tolerable levels of risk (Pooley et al. 2021). The implementation of prevention measures is pivotal to reducing carnivore impacts and fostering long‐term human‐carnivore coexistence in shared landscapes. Moreover, the involvement of local communities in carnivore monitoring is of paramount importance, as it serves the dual purpose of collecting reliable data and helping herders develop a more realistic perception of the actual impact carnivores have on livestock. Additionally, it plays a crucial role in increasing herders' sense of ownership, making them feel part of the solution rather than passive victims of carnivore presence. The role of herders as active decision‐makers warrants greater attention. Adoption and sustained use of deterrent devices are shaped not only by perceived effectiveness, but also by labor requirements, trust in conservation initiatives, prior depredation experience, and risk perception. Recognizing herders as agents within the system, rather than passive recipients of technical interventions, is essential for designing coexistence strategies that are socially legitimate and durable.

## Author Contributions

**Marcello Franchini:** data curation (equal), formal analysis (equal), investigation (equal), methodology (equal), validation (equal), visualization (equal), writing – original draft (equal), writing – review and editing (equal). **Charleen Gavette:** conceptualization (lead), funding acquisition (lead), resources (lead), supervision (equal), validation (equal), visualization (equal), writing – review and editing (equal). **Rodney Jackson:** conceptualization (lead), funding acquisition (lead), resources (lead), supervision (equal), validation (equal), visualization (equal), writing – review and editing (equal). **Kayley Bateman:** funding acquisition (lead), resources (lead), supervision (equal), validation (equal), visualization (equal), writing – review and editing (equal). **Ashleigh Lutz‐Nelson:** funding acquisition (lead), resources (lead), supervision (equal), validation (equal), visualization (equal), writing – review and editing (equal). **Geraldine Werhahn:** supervision (equal), validation (equal), visualization (equal), writing – review and editing (equal). **Yelik Nurbat:** data curation (equal), investigation (equal), validation (equal), visualization (equal), writing – review and editing (equal). **Marco Zaccaroni:** data curation (equal), validation (equal), visualization (equal), writing – review and editing (equal). **Fabio Dartora:** data curation (equal), validation (equal), visualization (equal), writing – review and editing (equal). **Munkhtsog Bayaraa:** validation (equal), visualization (equal), writing – review and editing (equal). **Claudio Augugliaro:** conceptualization (lead), data curation (equal), funding acquisition (lead), investigation (equal), methodology (equal), project administration (lead), resources (lead), supervision (equal), validation (equal), visualization (equal), writing – original draft (equal), writing – review and editing (equal).

## Funding

The project was funded by the Snow Leopard Conservancy.

## Ethics Statement

This study did not involve animal experimentation or invasive procedures on human participants. Participation by livestock herders was entirely voluntary and based on informed consent obtained prior to data collection. The purpose of the study, the voluntary nature of participation, and the intended use of the data for scientific research and publication were clearly explained to all respondents. All procedures were conducted in accordance with the ethical guidelines for research involving human participants and were approved by the Institutional Review Board (IRB) of the University of Udine (protocol no. 0068948), in compliance with the General Data Protection Regulation (EU) 2016/679. No personally identifiable or sensitive personal data were collected. All responses were anonymized prior to analysis, and no information enabling direct or indirect identification of individual respondents was retained. Given the nomadic lifestyle of herders, who relocate seasonally across different areas, the geographic location of individual gers does not allow for the identification of participants, either directly or indirectly.

## Conflicts of Interest

The authors declare no conflicts of interest.

## Supporting information


**Supporting Information: S1.** Track Survey Form.

**Supporting Information: S2.** Livestock depredations and prevention strategies to foster human–carnivore coexistence in Western Mongolia's high mountain systems.

**Figure S1:** Normal probability plot (QQ plot) of residuals of the Bayesian cumulative logit mixed‐effects model (BCMM).
**Figure S2:** Chains' trace plot of the Bayesian cumulative logit mixed‐effects model (BCMM).
**Figure S3:** Normal probability plot (QQ plot) of residuals of the first Bayesian generalized linear mixed model (BGLMM).
**Figure S4:** Chains' trace plot of the first Bayesian generalized linear mixed model (BGLMM).
**Figure S5:** Normal probability plot (QQ plot) of residuals of the second Bayesian generalized linear mixed model (BGLMM).
**Figure S6:** Chains' trace plot of the second Bayesian generalized linear mixed model (BGLMM).
**Figure S7:** Normal probability plot (QQ plot) of residuals of the Bayesian generalized linear model (BGLM).
**Figure S8:** Chains' trace plot of the second Bayesian generalized linear model (BGLM).

**Data S1:** ece374124‐sup‐0004‐supinfo‐dataset.xlsx.

## Data Availability

All the required data are uploaded as Supporting Information.
