# Supplementary material for: Livestock Depredations and Prevention Strategies to Foster Human‐Carnivore Coexistence in Western Mongolia's High Mountain Systems
Source: Ecol Evol. 2026 Jul 31;16(8):e74124. doi: 10.1002/ece3.74124 (PMC13427612; doi:10.1002/ece3.74124)
Supplement: Supplementary file 1 — Supporting Information: S1. Track Survey Form. [file ECE3-16-e74124-s001.docx]

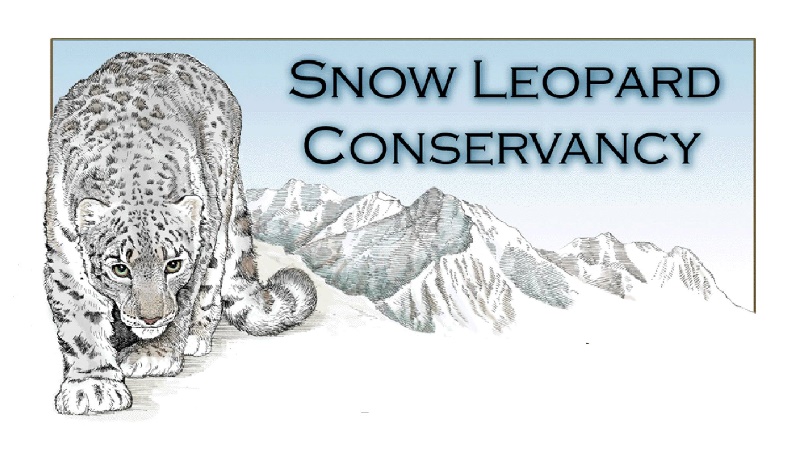

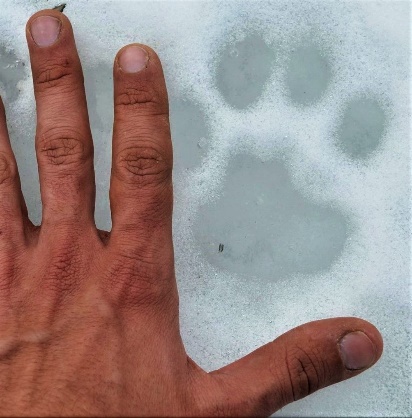


**Track Survey Form**

**Please fill out the form for each mid-large carnivore track you encounter, such as: wild or domestic animal depredation, sighting or footprint.**

ID________ (the ID must be filled by the interviewer before than deliver the forms, based on the corresponding ID in the questionnaire)

Date Obs. ____________ Coord. Obs.: Lon____________ Lat_______________

Locality ________________________ Observer (myself, relative) ___________

Estimated trail distance covered: ______________________km

Weather: _______ Time/Day of last snow fall: __________ Snow depth: __________cm

Track location / habitat (trees, shrubs, bare rocks / valley bottom, ridge, open space):

_________________________________________________________________________

Track Description

Carnivore Species___________ Number of individuals (based on sighting or footprints) __

Estimated track age (days or weeks): _________________

Toe-nail imprints (absent, present, uncertain): ______________________

Killing signs

Killed species: ___________ Age: _____ Number of individuals killed in the single event: ___________ Prey eaten parts: ________________________

Notes (i.e., drag marks, scats close to the prey, killing was in the night or daylight, other) ______________________________

_________________________________________________________________________
