# Supplementary material for: Livestock Depredations and Prevention Strategies to Foster Human‐Carnivore Coexistence in Western Mongolia's High Mountain Systems
Source: Ecol Evol. 2026 Jul 31;16(8):e74124. doi: 10.1002/ece3.74124 (PMC13427612; doi:10.1002/ece3.74124)
Supplement: Supplementary file 2 — Supporting Information: S2. Livestock depredations and prevention strategies to foster human–carnivore coexistence in Western Mongolia's high mountain systems. [file ECE3-16-e74124-s003.docx]

**Livestock depredations and prevention strategies to foster human-carnivore coexistence in Western Mongolia’s high mountain systems: Supplementary material 1**

The purpose of this questionnaire is to understand the opinion of livestock herders regarding the presence of large carnivores in the Bayan-Ölgii province. Additionally, we aim to explore their direct experiences with large carnivores in the area, particularly in relation to livestock predations, and the adopted livestock management systems to mitigate human-carnivore conflicts. The questionnaire is designed to take approximately 10 minutes to complete and is entirely anonymous to ensure confidentiality. Participation is voluntary, and all information provided will be used exclusively for research purposes by the authorized members of the study team. No data will be shared with unauthorized individuals. In accordance with ethical guidelines that protect the rights and privacy of research participants, no sensitive, personal, or confidential information will be collected. Participants are free to skip any question they do not wish to answer.

**A. RESPONDENT CARACHTERISTICS**

Respondent ID: ___________

Date: ___________

Longitude X: ___________

Latitude Y: ___________

1. Gender: M F

2. How old are you?

3. Since when do you live in this area (specify the province, sum district, bag district, Mountain, and valley name)?

4. How many people live currently in your house?

5. What is your level of education (i.e., years of study)?

6. What is your main job?

7. Do you have any other job?

**B. ADOPTED LIVESTOCK MANAGEMENT SYSTEM, LIVESTOCK LOSSES AND IMPLEMENTED PREVENTIVE MEASURES**

8. Who among you and your family member bring and guard the livestock to the grazing area?

9. How many livestock do you own (please specify the heads of each species)?

| Goats | Sheep | Horses | Yaks | Cattle | Camels |
| --- | --- | --- | --- | --- | --- |
|  |  |  |  |  |  |

10. How many days do you spend outdoor during a year?

11. Are snow leopard and wolf present in this area, according to your knowledge?

12. Do you have a corral? If yes during what period of the year you use it?

13. How many livestock do you lose due to disease, harsh winter, or other reason different than predation by carnivores?

14. How many head of each species you lost during last year due to the (carnivore’s name) (see the table below): goats___, sheep___, cow___, horses___, yak___, camels___? How many of these you lost during the night? Please specify when predated livestock is lamb, calf, foal.

| ***Interview ID*** | **predator species** | ***goat*** | ***sheep*** | ***cattle*** | ***horse*** | ***yak*** | ***camel*** |
| --- | --- | --- | --- | --- | --- | --- | --- |
|  |  |  |  |  |  |  |  |
|  |  |  |  |  |  |  |  |
|  |  |  |  |  |  |  |  |
|  |  |  |  |  |  |  |  |
|  |  |  |  |  |  |  |  |

15. Do you use a dog to protect your flock?

16. What species belong to your livestock are “free ranging”?

17. Do you use any method for deterring SL and wolf?

**C. ATTITUDE TOWARD SNOW LEOPARD AND WOLF**

18. **Please indicate your attitude towards following species (tick the category which most accurately describes your feeling toward the species in question):**

a. Snow leopard: Strongly dislike, Slightly dislike, Indifferent, Slightly like, Strongly like.

b. Wolf: Strongly dislike, Slightly dislike, Indifferent, Slightly like, Strongly like.

Thank you for your time!
