# Supplementary material for: Livestock Depredations and Prevention Strategies to Foster Human‐Carnivore Coexistence in Western Mongolia's High Mountain Systems
Source: Ecol Evol. 2026 Jul 31;16(8):e74124. doi: 10.1002/ece3.74124 (PMC13427612; doi:10.1002/ece3.74124)
Supplement: Supplementary file 3 — Figure S1: Normal probability plot (QQ plot) of residuals of the Bayesian cumulative logit mixed‐effects model (BCMM). Figure S2: Chains' trace plot of the Bayesian cumulative logit mixed‐effects model (BCMM). Figure S3: Normal probability plot (QQ plot) of residuals of the first Bayesian generalized linear mixed model (BGLMM). Figure S4: Chains' trace plot of the first Bayesian generalized linear mixed model (BGLMM). Figure S5: Normal probability plot (QQ plot) of residuals of the second Bayesian generalized linear mixed model (BGLMM). Figure S6: Chains' trace plot of the second Bayesian generalized linear mixed model (BGLMM). Figure S7: Normal probability plot (QQ plot) of residuals of the Bayesian generalized linear model (BGLM). Figure S8: Chains' trace plot of the second Bayesian generalized linear model (BGLM). [file ECE3-16-e74124-s002.docx]

**Livestock depredations and prevention strategies to foster human-carnivore coexistence in Western Mongolia’s high mountain systems: Supplementary material 3**


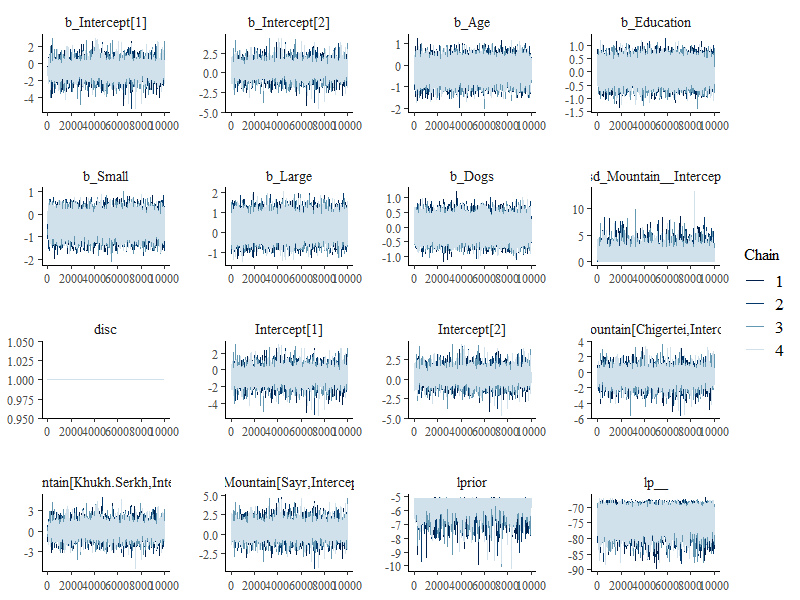


**Figure S1.** Normal probability plot (QQ plot) of residuals of the Bayesian cumulative logit mixed-effects model (BCMM).


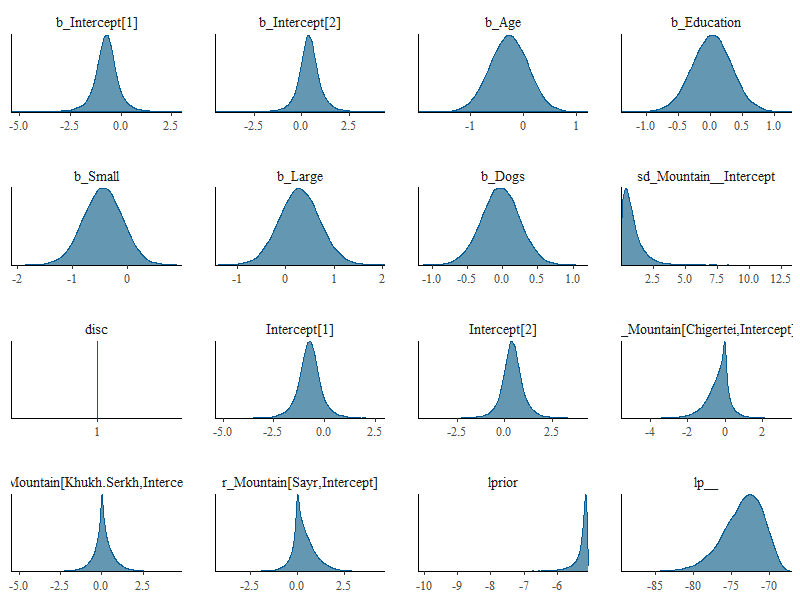


**Figure S2.** Chains’ trace plot of the Bayesian cumulative logit mixed-effects model (BCMM).


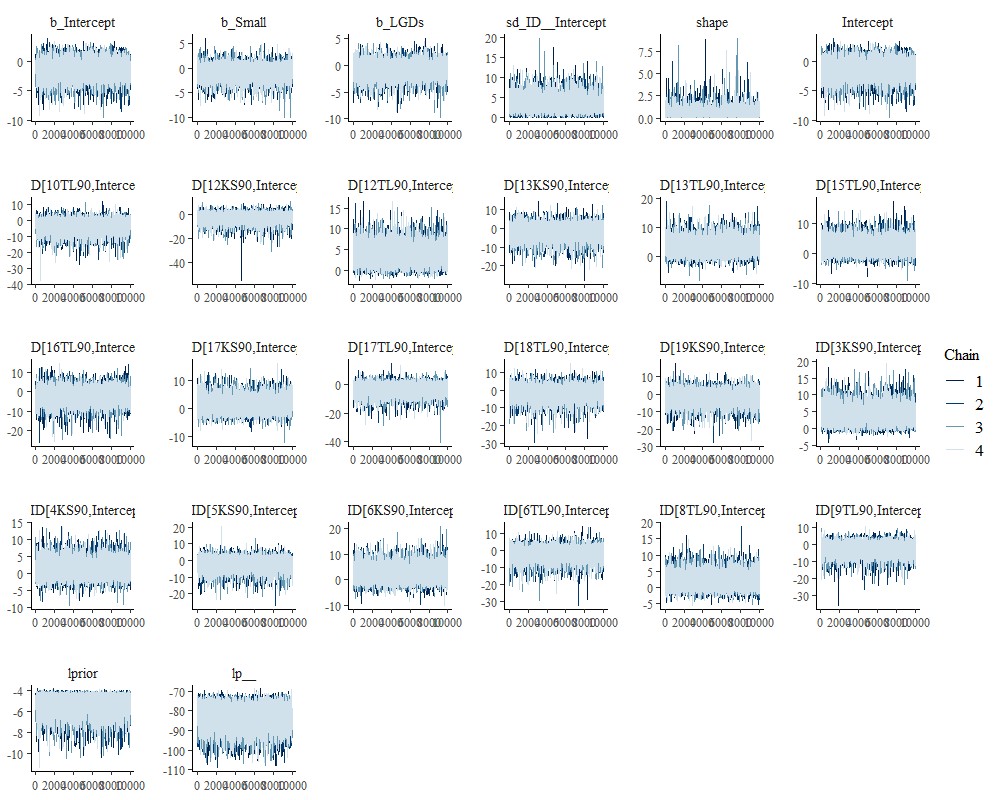


**Figure S3.** Normal probability plot (QQ plot) of residuals of the first Bayesian generalized linear mixed model (BGLMM).


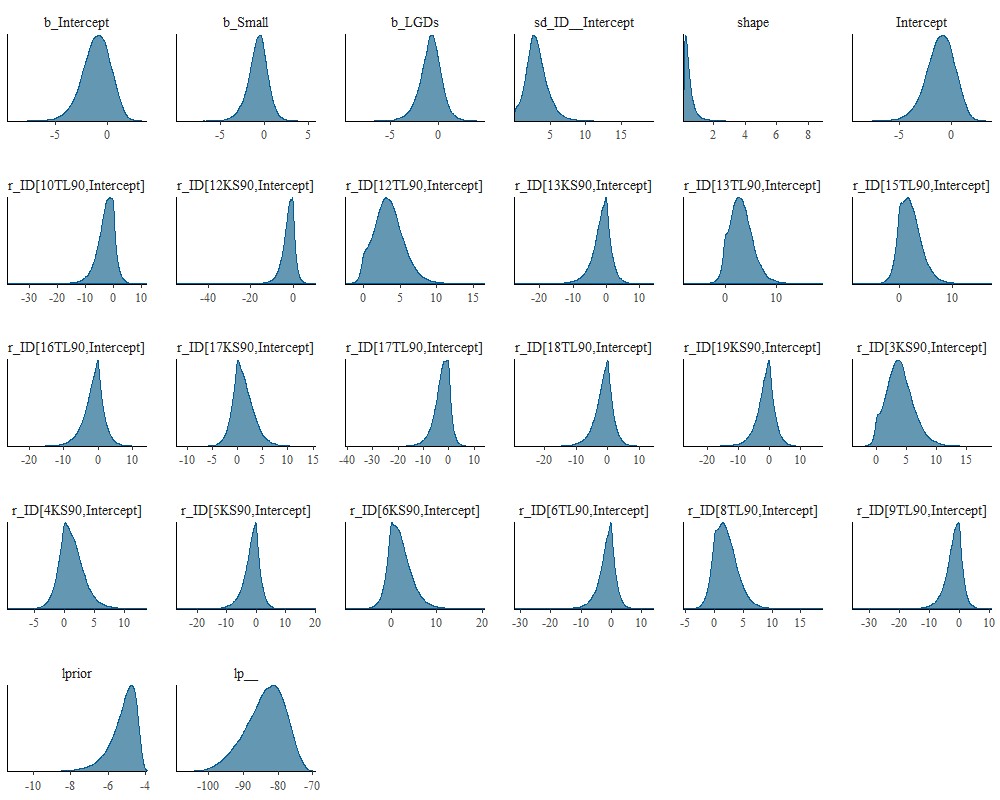


**Figure S4.** Chains’ trace plot of the first Bayesian generalized linear mixed model (BGLMM).


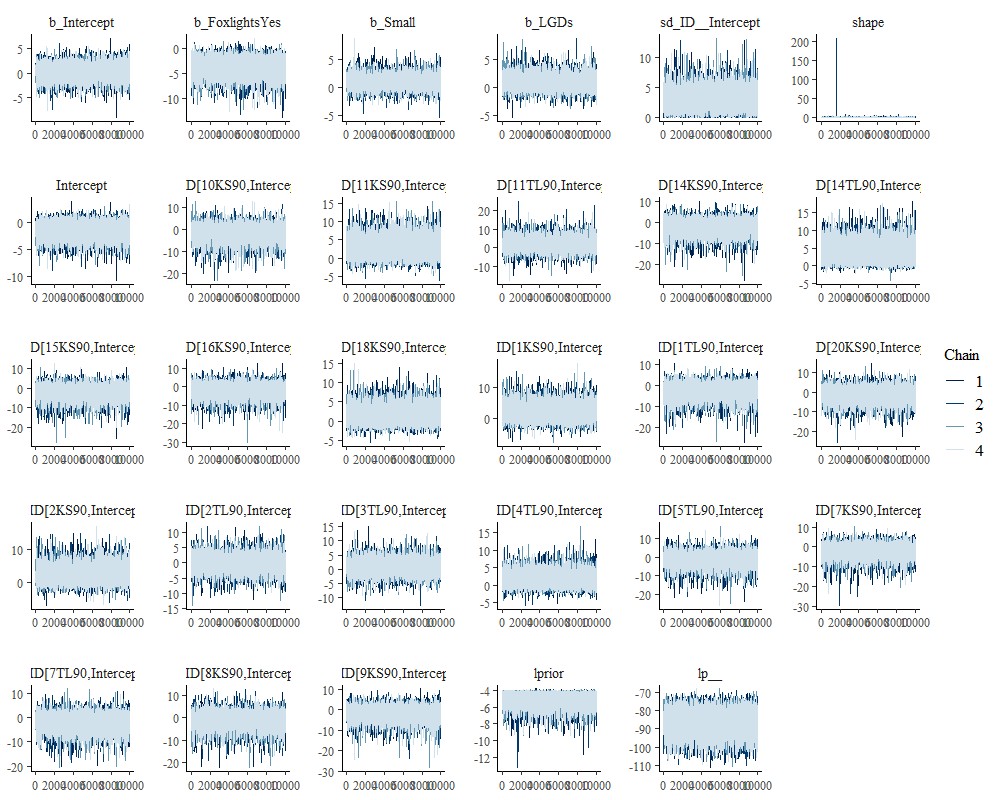


**Figure S5.** Normal probability plot (QQ plot) of residuals of the second Bayesian generalized linear mixed model (BGLMM).


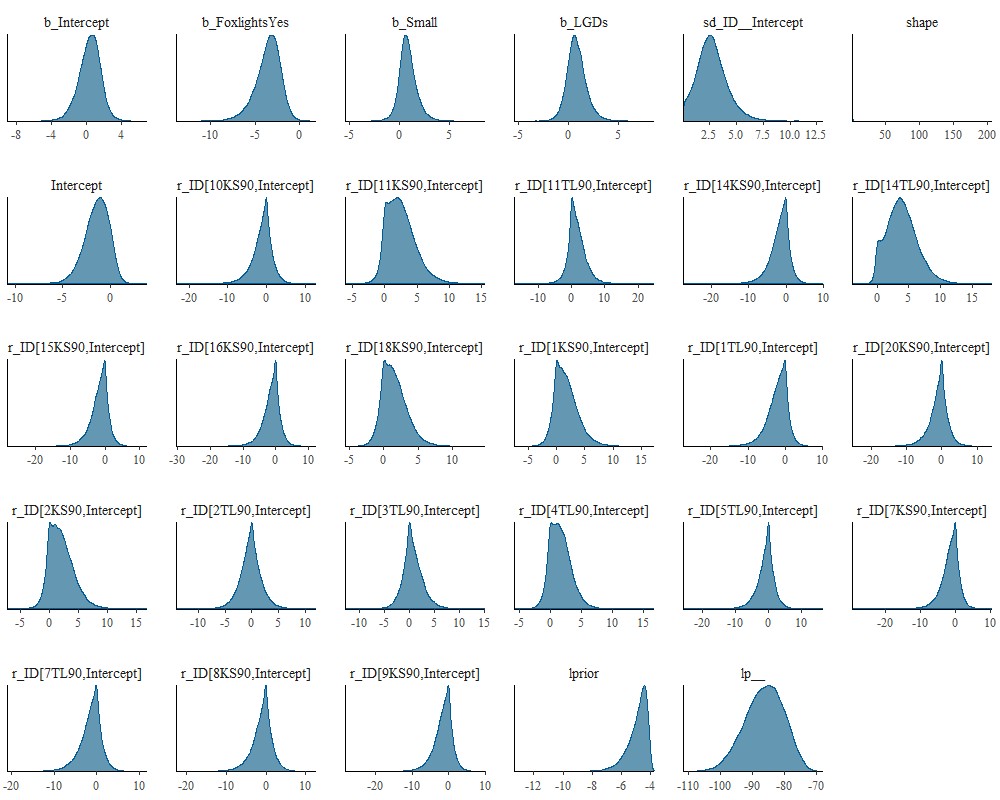


**Figure S6.** Chains’ trace plot of the second Bayesian generalized linear mixed model (BGLMM).


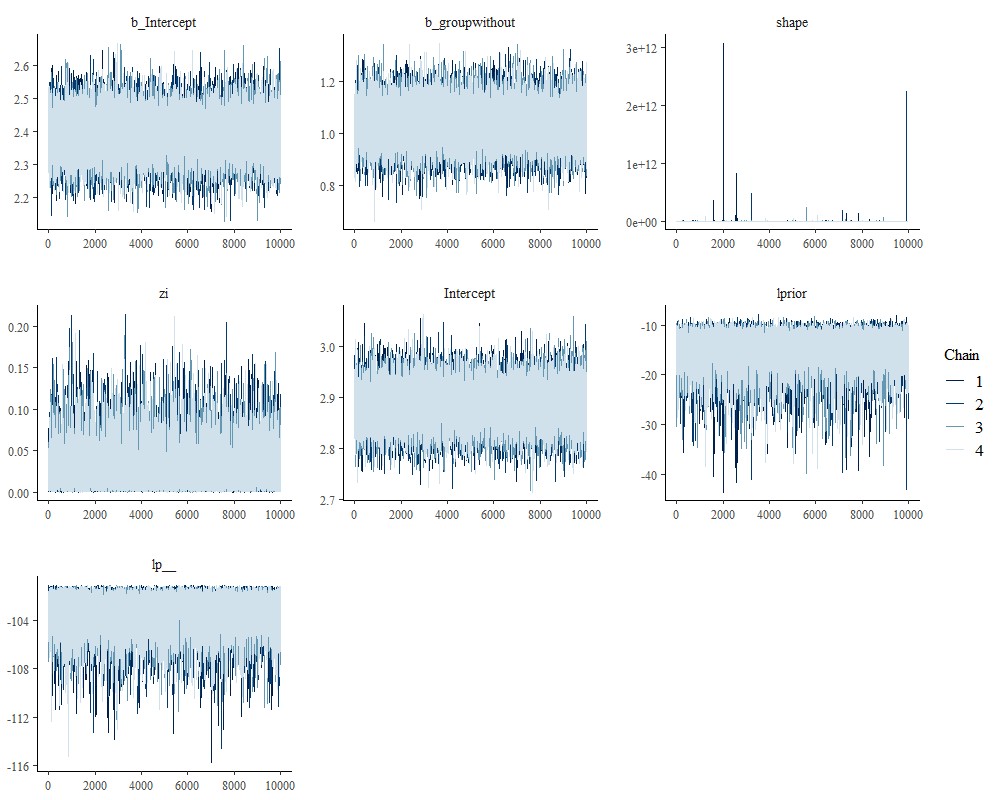


**Figure S7.** Normal probability plot (QQ plot) of residuals of the Bayesian generalized linear model (BGLM).


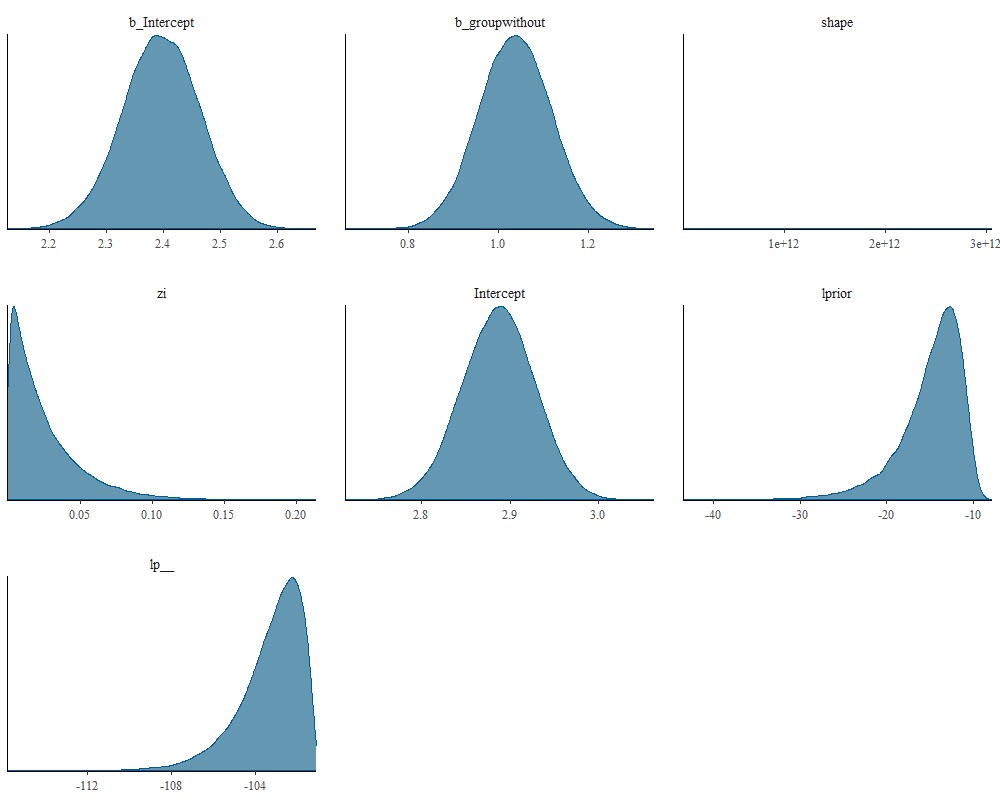


**Figure S8.** Chains’ trace plot of the second Bayesian generalized linear model (BGLM).
